# 7-Deazaguanine modifications protect phage DNA from host restriction systems

**DOI:** 10.1038/s41467-019-13384-y

**Published:** 2019-11-29

**Authors:** Geoffrey Hutinet, Witold Kot, Liang Cui, Roman Hillebrand, Seetharamsingh Balamkundu, Shanmugavel Gnanakalai, Ramesh Neelakandan, Alexander B. Carstens, Chuan Fa Lui, Denise Tremblay, Deborah Jacobs-Sera, Mandana Sassanfar, Yan-Jiun Lee, Peter Weigele, Sylvain Moineau, Graham F. Hatfull, Peter C. Dedon, Lars H. Hansen, Valérie de Crécy-Lagard

**Affiliations:** 1 Department of Microbiology and Cell Science, University of Florida, Gainesville, FL 32611 USA; 2 Department of Environmental Science, Aarhus University, Roskilde, Denmark; 3 Singapore-MIT Alliance for Research and Technology, Antimicrobial Resistance Interdisciplinary Research Group, Campus for Research Excellence and Technological Enterprise, Singapore, 138602 Singapore; 4 Department of Biological Engineering and Center for Environmental Health Sciences, Massachusetts Institute of Technology, Cambridge, MA 02139 USA; 5 School of Biological Sciences, Nanyang Technological University, 60 Nanyang Drive, Singapore, 637551 Singapore; 6 Département de Biochimie, Microbiologie et de Bio-informatique, Faculté des Sciences et de Génie, Université Laval, Québec City, QC G1V 0A6 Canada; 7 Félix d’Hérelle Reference Center for Bacterial Viruses and Groupe de Recherche en Écologie Buccale, Faculté de Médecine Dentaire, Université Laval, Québec City, QC G1V 0A6 Canada; 8 Pittsburgh Bacteriophage Institute and Department of Biological Sciences, University of Pittsburgh, Pittsburgh, PA 15260 USA; 9 Department of Biology, Massachusetts Institute of Technology, Cambridge, MA 02139 USA; 10 Research Department, New England Biolabs, Ipswich, MA 01938 USA; 11 University of Florida, Genetics Institute, Gainesville, Florida 32610 USA; 12 Present Address: Nitto Denko Avecia, 125 Fortune Boulevard, Milford, MA 01757 USA

**Keywords:** Biochemistry, Chemical biology, Computational biology and bioinformatics, Ecology, Evolution

## Abstract

Genome modifications are central components of the continuous arms race between viruses and their hosts. The archaeosine base (G^+^), which was thought to be found only in archaeal tRNAs, was recently detected in genomic DNA of *Enterobacteria* phage 9g and was proposed to protect phage DNA from a wide variety of restriction enzymes. In this study, we identify three additional 2′-deoxy-7-deazaguanine modifications, which are all intermediates of the same pathway, in viruses: 2′-deoxy-7-amido-7-deazaguanine (dADG), 2′-deoxy-7-cyano-7-deazaguanine (dPreQ_0_) and 2′-deoxy-7- aminomethyl-7-deazaguanine (dPreQ_1_). We identify 180 phages or archaeal viruses that encode at least one of the enzymes of this pathway with an overrepresentation (60%) of viruses potentially infecting pathogenic microbial hosts. Genetic studies with the *Escherichia* phage CAjan show that DpdA is essential to insert the 7-deazaguanine base in phage genomic DNA and that 2′-deoxy-7-deazaguanine modifications protect phage DNA from host restriction enzymes.

## Abstract (web summary)

Viral genomic DNA is often modified to evade the host bacterial restriction system. Here the authors identified 2′-deoxy-7-deazaguanine modifications on phage DNA by comparative genomics and experimental validation, showing their role in genome protection.

## Introduction

In the continuous battle between bacteria and phages, both entities are constantly evolving defenses and counterattack mechanisms^1–5^. To escape these defenses, phages have developed multiple strategies^6–8^, and one of the most widespread strategy is to modify their DNA. For example, the genomic DNA of *Escherichia coli* phage T4 contains the nucleobase glucosyl-hydroxymethylcytosine, which inhibits the restriction–modification (RM) and clustered regularly interspaced short palindromic repeat (CRISPR)–CRISPR-associated (Cas) systems^9^. The increased availability of complete phage genome sequences has led to recent discoveries of novel complex DNA modifications, such as 2′-deoxy-5-hydroxymethyluracil derivatives in *Pseudomonas* phage M6, *Salmonella* phage Vil, and *Deftia* phage phi W-14^10^ and 2′-deoxyarcheosine (dG^+^) in *Enterobacteria* phage 9g^11^.

**Fig. 1:**
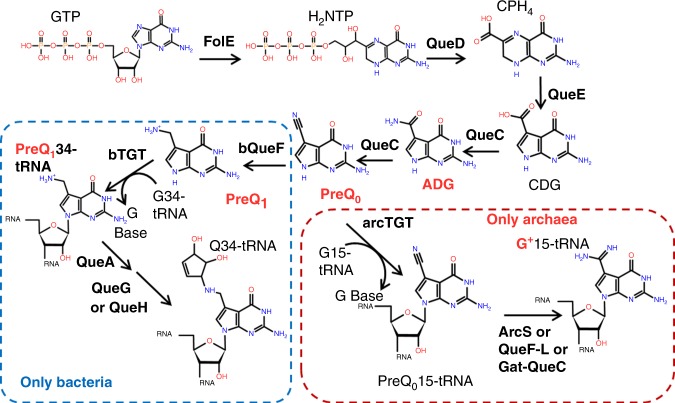
Queuosine and archeosine synthesis pathways. preQ_0_ is synthesized from GTP in both bacteria and archaea through FolE, QueD, QueE, and QueC, as shown. In most bacteria, four more enzymatic steps lead to the insertion of Q in tRNAs at position 34 (blue dashed square). In archaea, preQ_0_ is transferred to position 15 of tRNA before being modified to G^+^ (red dashed square). The bases found in phage DNA in this study are in red. Molecule abbreviations: guanosine tri-phosphate (GTP), dihydroneopterin triphosphate (H_2_NTP), 6-carboxy-5,6,7,8-tetrahydropterin (CPH_4_), 7-carboxy-7-deazaguanine (CDG), 7-amido-7-deazaguanine (ADG), 7-cyano-7-deazaguanine (preQ_0_), 7-aminomethyl-7-deazaguanine (preQ_1_), queuosine (Q), and archaeaosine (G^+^).

Two 7-deazaguanine modifications, 2′-deoxy-7-amido-7-deazaguanosine (dADG) and the 2′-deoxyribonucleoside analog of archaeosine, which were previously thought to be present only in tRNA as queuosine (Q) in bacteria and archaeosine (G^+^) in archaea, were recently discovered in bacteria and phage DNA, respectively, by combining in silico data mining and experimental validation^11^. As shown in Fig. 1, 7-cyano-7-deazaguanine (preQ_0_) is synthesized from GTP by four enzymes (FolE, QueD, QueE, QueC) and is the key intermediate in both the Q and G^+^ pathways^12–14^. tRNA-guanine-transglycosylases (TGT in bacteria, arcTGT in archaea) are the signature enzymes in the Q and G^+^ tRNA modification pathways, as they exchange the targeted guanines with 7-deazaguanine precursors. In archaea, preQ_0_ is directly incorporated into tRNA by arcTGT before being further modified by different types of amidotransferases (ArcS, Gat-QueC, or QueF-L)^15–17^. In bacteria, preQ_0_ is reduced to 7-aminomethyl-7-deazaguanine (preQ_1_) by QueF^18^ before TGT incorporates it in tRNA^19^, where it is further modified to Q in two steps^20–22^ (Fig. 1).

The presence of homologs of Q synthesis genes has long been reported in phage genomes^23–26^. However, the role of these genes in DNA modification rather than in RNA modification was only recently postulated. Indeed, TGT paralogs (now called DpdA) were found to be involved in modifying DNA in specific bacteria and phage genomes. In bacteria, the *dpdA* gene is often located in a cluster of over ten genes that encode a RM system that inserts ADG into DNA and prevents replication of unmodified DNA^11,27^. In *Enterobacteria* phage 9g^28^, *dpdA* is associated with G^+^ synthesis, and up to 27% of the dG in this phage is replaced by dG^+^^11,29^. This modification is proposed to play an anti-restriction role^28^ because 7-deazaguanine derivatives can block the activity of a wide variety of restriction enzymes without inhibiting the activity of the polymerases needed for phage DNA replication^30^.

Building on the discovery of dG^+^ in *Enterobacteria* phage 9g, we systematically explore the genomes of other phages for potential pathways involved in 7-deazaguanine insertion in DNA and experimentally validate a subset. This work reveals a much greater diversity in the 7-deazaguanine modifications and their corresponding pathways than anticipated. Moreover, we show that 7-deazaguanine derivatives have been hijacked by phages to evade RM systems.

## Results

### Phage 9g encodes functional preQ_0_ synthesis genes

**Fig. 2:**
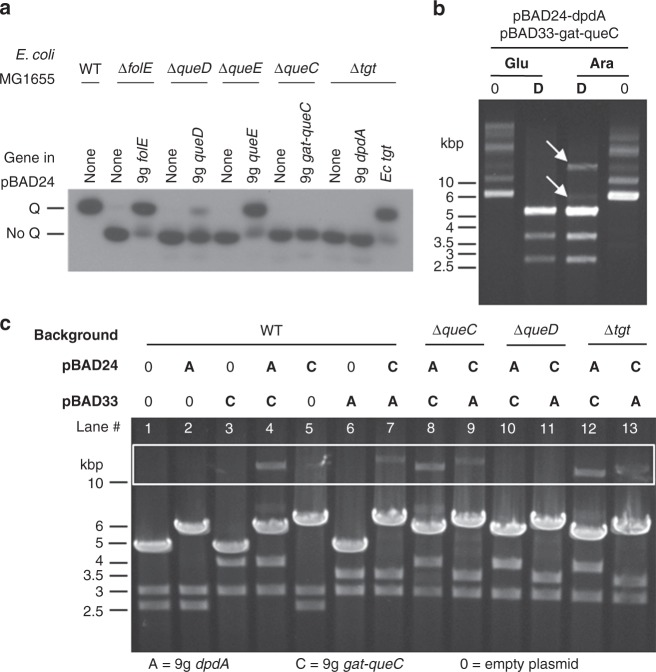
In vivo activity tests for *Enterobacteria* phage 9g dG^+^ pathway genes. Source data are provided as a Source Data file. **a** Northern blot of an acrylamide electromobility gel shift assay showing the tRNA-Q complementation of *E. coli* mutants by *Enterobacteria* phage 9g orthologs. The WT strain modifies tRNA_Asp_ with Q and is shifted in its migration (Q line), but the *E. coli* mutant strains (Δ*folE*, Δ*queD*, Δ*queE*, Δ*queC*, and Δ*tgt*) are not modified and migrate further (no Q line). In each mutant, the orthologs of *Enterobacteria* phage 9g are expressed in *trans*. The complementation of Δ*tgt* by *E. coli tgt* is shown as a positive control of complementation. **b** Agarose gel of uncut (0) or EcoRI-cut (D) pGH39/pGH66 extracted from a WT strain of *E. coli*; expression from the plasmids was repressed in 0.4% glucose (Glu) or induced in 0.4% arabinose (Ara). White arrows indicate the undigested plasmids. **c** Agarose gel of EcoRI digestion of plasmids extracted from different strains of *E. coli* (WT, Δ*queC*, Δ*queD*, Δ*tgt*) carrying variants of pBAD33 and pBAD24 (empty plasmid, 0; encoding *Enterobacteria* phage 9g *dpdA*, A; or encoding *Enterobacteria* phage 9g *gat-queC*, C). EcoRI cuts pBAD24 once (4542-bp fragment) and pBAD33 twice (2479 and 2873-bp fragments). The resulting sizes for the digestion of pBAD24 are 5971 and 5509 bp when *gat-queC* or *dpdA* is inserted, respectively. For pBAD33, the 2873-bp fragment remains unchanged, but the 2479-bp fragment shifts to 3911 when *gat-queC* is inserted and 3449 bp when *dpdA* is inserted. When plasmids are undigested, they can be seen in the white rectangle zone.

The expression of *folE*, *queD*, and *queE* from *Enterobacteria* phage 9g in *trans* in *E. coli* MG1655 Δ*folE*, Δ*queD*, and Δ*queE* strains, respectively, successfully re-established the production of Q, demonstrating the isofunctionality of the tested pairs (Fig. 2a). This complementation was not observed when the viral *gat-queC* and *dpdA* genes were expressed in *E. coli* Δ*queC* and Δ*tgt*, respectively. The result was expected for *dpdA*, as *dpdA* was predicted to encode an enzyme that recognizes DNA and not tRNA^11,31^. This result was unexpected for *gat-queC*, as we had previously shown that expression of an archaeal *gat-queC* homolog in *E. coli* could lead to G^+^ in tRNA and hence the formation of a preQ_0_ intermediate^16^.

### Phage 9g Gat-QueC and DpdA insert G^+^ DNA

As *E. coli* encodes the entire preQ_0_ biosynthesis pathway, we predicted that the dual expression of the viral *gat-queC* and *dpdA* genes in *trans* would lead to the insertion of 7-deazaguanine derivatives, such as dG^+^, in *E. coli* DNA. Because the presence of dG^+^ confers resistance to EcoRI digestion^29^, we used restriction profiles as a first indication for the presence of modifications in plasmid DNA. The two phage genes were both cloned into pBAD24 and pBAD33. EcoRI cuts pBAD24 once and pBAD33 twice, as shown in the digestion profiles of plasmids extracted from *E. coli* cotransformed with the two empty plasmids (Fig. 2b, c, lane 1). Because the *gat-queC* and *dpdA* genes of phage 9g lack EcoRI sites, the restriction profiles of plasmids extracted from *E. coli* derivatives cotransformed with an empty plasmid and a plasmid containing one of the two genes are shifted by the insert sizes (Fig. 2c, lanes 2, 3, 5, and 6). An additional band corresponding to the uncut plasmid was observed only for plasmids extracted from strains expressing both *gat-queC* and *dpdA* genes (Fig. 2c, lanes 4 and 7, and Fig. 2b, white arrows). As a supplemental control, we digested the same combination of plasmids with PsiI (TTA^TAA) and EcoRI (Supplementary Fig. 1). The single digestion by PsiI linearized all these plasmids, and the plasmids encoding both *dpdA* and *gat-queC* of phage 9g were again partially resistant to EcoRI digestion (red arrows in Supplementary Fig. 1).

Analysis of dG^+^, dADG, dPreQ_0_, and dPreQ_1_ profiles by liquid chromatography-coupled triple quadrupole mass spectrometry (LC-MS/MS, quantification results in Table 1, mean ± standard deviation based on two or three replicates) revealed that plasmid DNA extracted from strains expressing only *dpdA* contained dPreQ_0_, with 790 ± 8 modifications per 10^6^ nucleotides; 0.316 ± 0.0032% of the Gs, when expressed in pBAD24; and 84 ± 26 modifications per 10^6^ nucleotides, 0.0336 ± 0.0104% of the Gs, when expressed in pBAD33. dG^+^ was detected in this strain just above the detection limit as well (6.5 ± 0.5 modifications per 10^6^ nucleotides, 0.0026 ± 0.0002% of the Gs). Plasmid DNA extracted from strains expressing *dpdA* and *gat-queC* contained dG^+^, with 45,000 ± 25,000 modifications per 10^6^ nucleotides, 18 ± 10% of the Gs, when DpdA was expressed in pBAD24 and Gat-QueC was expressed in pBAD33 and 22,750 ± 17,250 modifications per 10^6^ nucleotides, 9.1 ± 7% of the Gs, when reversed. dPreQ_0_ was also detected when *gat-queC* was expressed at lower levels than *dpdA*, (77 ± 7 modifications per 10^6^ nucleotides, 0.0308 ± 0.0028% of the Gs). No modifications were detected in strains harboring empty plasmids or when only Gat-QueC was expressed (Table 1). Taken together, these results showed that dG^+^ but not preQ_0_ confers resistance to EcoRI and that the phage 9g pathway that inserts dG^+^ in its viral DNA can be transferred to modify *E. coli* genomic DNA.

**Table 1:** DNA modifications identified by mass spectrometry in the plasmids shown Fig. 2b.

Lane in Fig. 2b	Background	9g gene in pBAD24	9g gene in pBAD33	*dADG* per 10^6^ nt	dPreQ_0_ per 10^6^ nt	dPreQ_1_ per 10^6^ nt	*dCDG* per 10^6^ nt	dG^+^ per 10^6^ nt
1	MG1655	None	None	<6	<6	<6	<6	<6
2	MG1655	*dpdA*	None	<6	790 ± 8	<6	<6	<6
3	MG1655	None	*gat-queC*	<6	<6	<6	<6	<6
4	MG1655	*dpdA*	*gat-queC*	<6	77 ± 7*	<6	<6	45,000 ± 25,000
5	MG1655	*gat-queC*	None	<6	<6	<6	<6	<6
6	MG1655	None	*dpdA*	<6	84 ± 26	<6	<6	6.5 ± 0.5
7	MG1655	*gat-queC*	*dpdA*	<6	<6	<6**	<6**	22,750 ± 17,250
8	MG1655 Δ*queC*	*dpdA*	*gat-queC*	<6**	<6**	<6**	<6**	13,750**
9	MG1655 Δ*queC*	*gat-queC*	*dpdA*	<6	<6	<6	<6	23,000 ± 17,000

All values represent the mean ± deviation of the mean for two analyses, except asterisk (*), mean ± standard deviation for three replicate analyses, and double asterisks (**), single analysis

Interestingly, whereas we had failed to complement the Q^−^ phenotype of the *E. coli ΔqueC* strain when expressing the *gat-queC* gene of phage 9g, the EcoRI resistance phenotype caused by 7-deazaguanine insertion in strains expressing both *dpdA* and *gat-queC* of phage 9g was still observed in a *ΔqueC* background (Fig. 2c, lanes 8 and 9) but not in a *ΔqueD* background (Fig. 2c, lanes 10 and 11). Furthermore, only dG^+^ modification was observed in the DNA of the *ΔqueC* strains by LC-MS/MS (Table 1), with similar amounts as in the wild type (WT; 13,750 modifications per 10^6^ nucleotides, 5.5% of the Gs, and 23,000 ± 17,000 modifications per 10^6^ nucleotides, 9.2 ± 7% of the Gs). This suggests that the Gat-QueC protein can produce preQ_0_ but that it is channeled to the putative DNA-modifying enzyme DpdA and not to the tRNA-modifying pathway enzyme QueF.

Finally, we tested whether the *E. coli* TGT was required for DpdA activity in *E. coli*, as the active forms of TGT enzymes are known to be dimers^31^. This did not seem to be the case, as the restriction resistance phenotype was still observed in the Δ*tgt* background (Fig. 2c, lanes 12 and 13).

### A wide variety of phages encode dG^+^ synthesis proteins

We identified another subfamily of DpdA, renamed DpdA2, encoded by the *Vibrio* phage nt-1 by investigating genes flanking the preQ_0_ biosynthesis gene cluster. Indeed, DpdA2 (YP_008125322) of phage nt-1 is not detected when using *Enterobacteria* phage 9g DpdA as a query in PSI-BLAST. This DpdA2 family does not possess the conserved histidine found at position 196^11^. However, some similarities with members of the TGT family were detected using HHpred, with a confidence score of 100%.

An in silico search for phages that could harbor 7-deazaguanine derivatives in their genomic DNA revealed a total of 182 viruses deposited in GenBank that were found to encode a DpdA/DpdA2 homolog and/or at least a G^+^ synthesis gene (Supplementary Data 1). Most of these viruses (163/182) were bacteriophages, while 16 were archaeal viruses and 3 were eukaryotic viruses. The eukaryotic viruses only encode FolE, which is most likely linked to the folate pathway^32^. Analyses of the presence/absence patterns of the predicted Q/G^+^ biosynthesis genes led to a classification of these viruses into various groups and, in some cases, predicted the nature of the 7-deazaguanine base modification. It is important to note that no homologs to the proteins specifically involved in Q biosynthesis, such as QueA, QueG, or QueH (see Fig. 1), were found in the viruses analyzed.

**Fig. 3:**
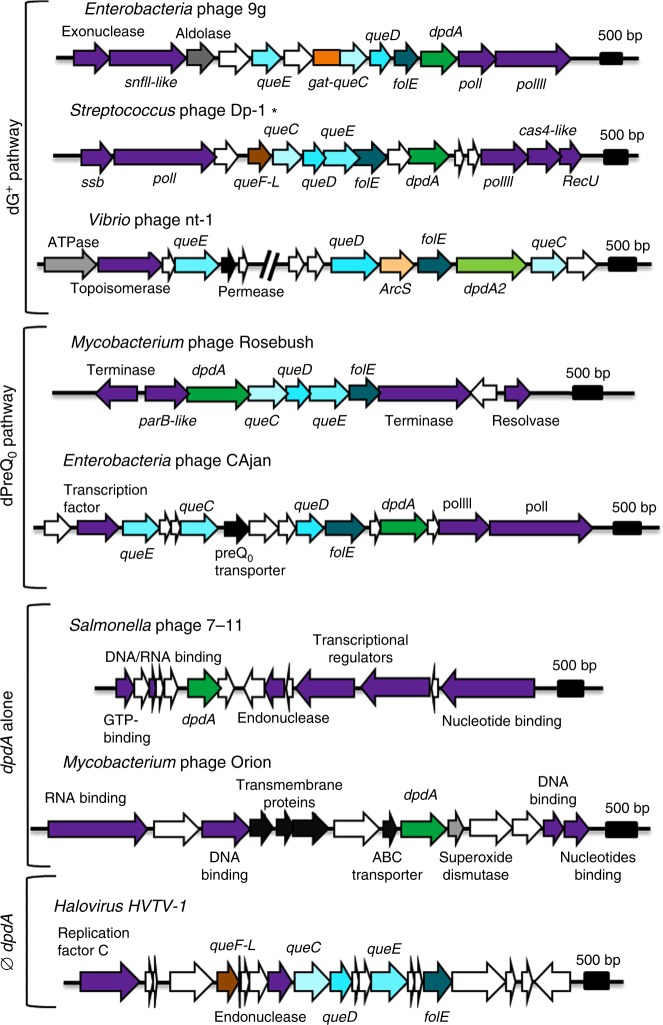
Genomic context of the *dpdA* and dG^+^/preQ_0_ biosynthesis pathway genes. *Enterobacteria* phage 9g, *Streptococcus* phage Dp-1, *Vibrio* phage nt-1, *Mycobacterium* phage Rosebush, *Escherichia* phage CAjan, *Salmonella* phage 7–11, *Mycobacterium* phage Orion, and Halovirus HVTV-1 are presented. The genes are colored by functions: green is DpdA, shades of blue are the biosynthetic pathway of preQ_0_, shade of oranges are the genes coding for aminotransferases that synthetize G^+^ from preQ_0_, red is QueF. In purple are the proteins involved in DNA metabolism; in black, the transmembrane proteins; in gray, all other known functions; and in white, genes coding for unknown functions. Asterisk (*): Note that we grouped *Streptococcus* phage Dp-1 in the dG^+^ biosynthesis pathway in our bioinformatics analysis, but it does not produce this modification. Source data are provided as a Source Data file.

The first group contains 25 phages and is represented by *Enterobacteria* phage 9g (KJ419279), *Streptococcus* phage Dp-1 (NC_015274), and *Vibrio* phage nt-1 (NC_021529) in Fig. 3. These phages encode homologs of 9g DpdA or nt-1 DpdA2 as well as of FolE, QueD, QueE, and QueC. In addition, they encode homologs of one of the three amidotransferases involved in the last steps of G^+^ synthesis: ArcS^15^, QueF-L^16^ (or QueF), or a glutamine amidotransferase (Gat) domain fused to the canonical QueC^16^. These phages likely modify their DNA with dG^+^, as does phage 9g^11^. It should be noted that the discrimination between the QueF-L homologs, predicted to produce the G^+^ base from preQ_0_, and QueF homologs, predicted to produce preQ_1_ from preQ_0_, is difficult to establish based only on sequence similarity. Therefore, the phages encoding these proteins might harbor dG^+^ or dPreQ_1_ (or both). Of note, this viral group includes a *Pseudomonas aeruginosa* phage that was isolated; the genome of this phage was sequenced in this study, and the phage was named *Pseudomonas* phage Quinobequin P09 (description in Supplementary Information).

The second group includes 40 phages and is represented by *E. coli* phage CAjan (NC_028776) and *Mycobacterium* phage Rosebush (AY129334) in Fig. 3. These phages encode a homolog of one of the two types of DpdA and of the preQ_0_ synthesis enzymes (FolE, QueD, QueE, and QueC), but they are missing an amidotransferase. As such, we predicted that these phages modify their DNA with preQ_0_ or ADG, similar to the bacteria that contain the *dpd* cluster^11^. *Mycobacterium* phage Bipper (KU728633), which is only missing a gene coding for QueC, was added to this group even if it could be modified by the QueC substrate (7-carboxy-7-deazaguanine, see Fig. 1). The Uncultured phage clone 7AX_2 (MF417872) was also added to this group because it lacks *queC*, although this may be due to the incomplete genome sequence of this phage. In addition, we cannot exclude that this phage encodes an amidotransferase.

The third group is currently the largest, as it contains 76 phages, including *Salmonella* phage 7–11 (NC_015938) and *Mycobacterium* phage Orion (DQ398046), as shown in Fig. 3. These phages encode DpdA but no G^+^ or preQ_0_ biosynthesis protein homologs. At this stage, their genome modification status, if any, is difficult to predict. Phages in this group could rely on preQ_0_ synthesized by the host or on the uptake of exogenous 7-deazapurine precursors. Some phages do encode homologs of YhhQ, the preQ_0_ transporter^33^, but there is no correlation with any specific group of phages. The large size of this group compared to the others might be caused by the relatively large number of Mycobacteriophages in the Virus database due to the massive phage isolation and sequencing effort of PhagesDB and the SEA-PHAGES project^34^.

The last group is composed of 48 phages encoding proteins of the preQ_0_/G^+^ pathway but not DpdA. These phages could boost the production of the Q precursor to increase the level of Q in the host tRNA and increase translation efficiency^35^. However, it is possible that 7-deazaguanines are inserted in their DNA in a DpdA-independent pathway, as there is a recent report that the genomes of *Campylobacter* phages of this group are highly modified by dADG^36^. Similarly, the Halovirus HVTV-1 (NC_020158), presented in Fig. 3, may have found another way to insert the modifications and should harbor either dPreQ_1_ or dG^+^, as it encodes the QueF, or QueF-like, protein.

Phages containing FolE and QueC singletons were discarded from further analysis because FolE is shared between folate and preQ_0_ synthesis^13^, while QueC is also part of a superfamily of ATPases^37^, making their precise role difficult to identify.

All the phages identified above are members of the *Caudovirales* order and are distributed into various families: *Siphoviridae* (95), *Myoviridae* (23), *Ackermannviridae* (20), and *Podoviridae* (3). For the Archaeal viruses, we identified 12 members of the *Ligamenvirales* order and 2 of the *Bicaudaviridae* family (Supplementary Data 2).

### Detailed analysis of phage 7-deazaguanine synthesis proteins

To evaluate the isofunctionality of the studied protein families, sequence similarity networks (SSNs) were generated. Proteins in the same cluster should share the same function^38^. Several of the 7-deazaguanine biosynthesis proteins are part of protein families that are known to harbor subgroups with different functions that could impede functional annotations using only PSI-BLAST scores or HMM models, hence the use of SSNs to strengthen the annotation process.

**Fig. 4:**
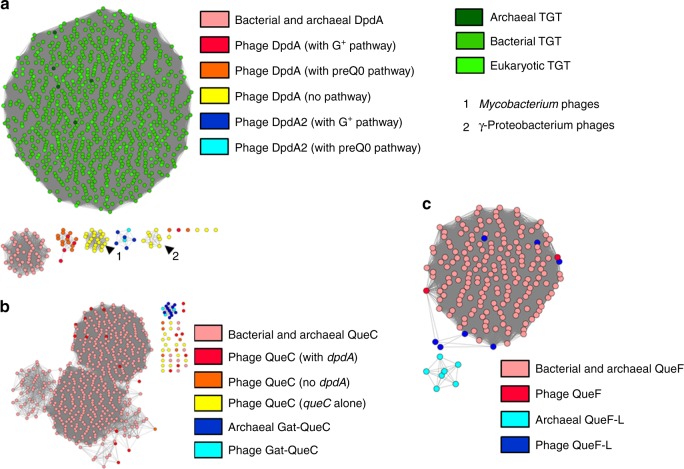
Protein similarity networks. Source data are provided as a Source Data file. **a** DpdA/Tgt protein network, each node is a group of proteins identical at 90%, and each edge presents an alignment score >15. The TGTs of archaea, bacteria, and eukaryotes are shown in dark green, green, and light green, respectively. Bacterial DpdA are shown in light red. The phage DpdA are separated depending on the gene content of phages: in red, DpdA in genomes encoding the G^+^ pathway; in orange, the preQ_0_ pathway; in yellow, the genomes with only *dpdA*; in dark blue, DpdA2 with G^+^ pathway; and in light blue, DpdA2 with a preQ_0_ pathway. The arrow shows clusters of nodes specific to a clade of a bacterial host (1 is *Mycobacterium* and 2 is γ-*Proteobacteria*). **b** QueC protein network, with a threshold alignment score of 44. In light red, the QueC from bacteria; in dark red, the QueC from phages that encode a DpdA; in orange, the QueC from phages that are not encoding a DpdA; and in yellow, the QueC from phage encoding only a QueC. Gat-QueC from archaea is in dark blue and from phages is in light blue. **c** QueF protein network with an alignment score threshold of 10. In light red, the bacterial QueF; and in dark red, the protein identified as phage QueF. In light blue, the archaeal QueF-L; and in dark blue, the phage protein identified as QueF-L.

As shown in Fig. 4a, phage DpdA proteins do not cluster with the TGT proteins from the three major kingdoms nor with the bacterial DpdA proteins identified previously^11^. Phage DpdA clearly separate in four subgroups. One contains the DpdA found in phages that encode the complete set of G^+^ or preQ_0_ synthesis proteins. The second and third groups are composed of singleton DpdA proteins, and the fourth group is composed of DpdA2 proteins. The singleton DpdAs are clustered in phages that infect the same clade of bacteria (*Mycobacterium* and γ-*Proteobacteria*). This could be a sign of a rapid divergence of this protein subfamily, and more studies will be required to determine whether this subset of DpdA proteins has functionally diverged.

Most phage QueC proteins do not cluster with bacterial QueC proteins when the BLAST threshold score is sufficient to separate QueC from the Gat-QueC groups (Fig. 4b). However, when a lower threshold score is used, the QueC and Gat-QueC proteins can be connected (Supplementary Fig. 2A). This is not the case for the QueC proteins encoded as singletons in phages, such as *Bacillus* phage SP-15 and *Salmonella* phage SFP10 (Supplementary Data 1), suggesting that even though the proteins were identified as QueC by HHpred, they may be part of a functionally unrelated subgroup of the N-type ATP pyrophosphatases superfamily^37^. Finally, phage and archaeal Gat-QueC proteins form a single cluster, strengthening their functional association.

HHpred predicted that the QueF family proteins encoded by phages are, for most of them, closer to the archaeal QueF-L proteins than to the bacterial QueF proteins (see Supplementary Data 1). However, they clustered with bacterial QueF proteins in the SSNs (Fig. 4c). Further experimental studies are required to determine whether the phage QueF proteins are nitrile reductases or amidotransferases (Fig. 1).

SNNs for the FolE, QueD, QueE, and ArcS families are shown in Supplementary Fig. 2B–E. The phage proteins cluster nicely with their bacterial and archaeal homologs, reinforcing the initial functional annotations.

### The host may participate in phage DNA modification

To study the interaction between phages containing 7-deazaguanine-related genes and their bacterial hosts, we gathered metadata on the hosts and their habitat using RefSeq^39^ and the Globi database^40^ and analyzed the distribution of Q, G^+^, and dADG synthesis genes in these organisms (see Supplementary Data 2 and 3). Interestingly, 106 of the collected phages (~60%) infect a host strain that is the model for a known bacterial pathogen (Supplementary Data 2), where only ~9% of all the double-stranded DNA (dsDNA) viruses from the Virus-Host database^41^ infect a strain related to pathogens (data not shown), making our sample six to seven times more enriched compared to a random sampling. No clear environment was found for the archaeal hosts.

All phage hosts predicted to modify their DNA with G^+^ possess the pathway to produce Q in tRNA. Curiously, the hosts of phages coding for a QueF-L and a 9g DpdA homolog do not encode the preQ_0_ biosynthetic pathway (QueDEC, see Fig. 1) but encode the specific preQ_0_ transporter YhhQ^33^ and the rest of the Q pathway (QueFAG and TGT, Fig. 1). Conversely, all the hosts of the DpdA2-encoding phages encode the full Q pathway.

There is no clear pattern for the bacterial hosts of phages encoding both DpdA and the whole preQ_0_ pathway. Most of them encode the full Q pathway enzymes except for *Streptococcus pneumoniae*, which lacks the preQ_0_ pathway genes; *Rhodococcus erythropolis*, which encodes only TGT; and *Mycobacteria*, which possess none of these genes.

The hosts of the phages encoding only DpdA also encode the full set of Q synthesis enzymes except the *Clostridium* species, which lack the preQ_0_ pathway genes, and the *Mycobacterium* genus, which possesses none of these genes. *Sulfolobi* were not referenced in PubSEED^42^, but by performing a BLASTp search with default parameters and the genes listed in Supplementary Table 1 as queries, we identified all G^+^ pathway genes (Supplementary Table 2). Hence, the 7-deazaguanine intermediates produced by these hosts, *Clostridium* and *Mycobacterium* excluded, might be used by phages that lack the biosynthesis proteins to produce a 7-deazaguanine precursor.

Finally, the hosts of the phages that do not encode a DpdA homolog but encode the preQ_0_ pathway proteins all encode the full Q synthesis pathway.

A few bacterial hosts, such as 46 different strains of *E. coli*, *Haloarcula vallismortis*, and *Vibrio harveyi* 1DA3, also harbor homologs of the bacterial DpdA, which are known to modify bacterial DNA by either dPreQ_0_ or dADG^11^.

### Different 7-deazaguanine modifications in distinct phages

To test our predictions on the nature of phage DNA modifications, a set of phages from each group were selected (Fig. 3), and their genomic DNAs were extracted for mass spectrometric analysis (Table 2, mean ± standard deviation based on two replicates). No 2′-deoxyqueuosine (dQ) was found in any of the tested samples, correlating with the fact that no phage or virus encodes the specific protein for Q synthesis (QueAGH).

**Table 2:** DNA modifications identified by mass spectrometry in the different phages.

Phage/virus Accession #	Phage/virus name	Phage/virus GC content	Prediction based on gene content	dPreQ_0_ per 10^6^ nt	dADG per 10^6^ nt	dG^+^ per 10^6^ nt	dPreQ_1_ per 10^6^ nt	dQ per 10^6^ nt
NC_028776	*Escherichia* phage CAjan	44.70%	dPreQ_0_	70,628 ± 2445	<6	<6	<6	<6
None	*Escherichia* phage CAjan Δ*dpdA*		None	<6	<6	<6	<6	<6
NC_020158	Halovirus HVTV-1	58.30%	None/dG^+^	<6	152 ± 3	22 ± 1	88,607 ± 3014	<6
NC_008197	*Mycobacterium* phage Orion	66.50%	None	<6	<6	<6	<6	<6
NC_004684	*Mycobacterium* phage Rosebush	69.00%	dPreQ_0_	96,530 ± 2529	9 ± 1	<6	<6	<6
NC_015938	*Salmonella* phage 7–11	44.10%	None/PreQ_0_	<6	50 ± 2	<6	<6	<6
NC_015274	*Streptococcus* phage Dp-1	40.30%	dPreQ_1_/dG^+^	<6	<6	<6	3389 ± 184	<6
NC_021529	*Vibrio* phage nt-1	41.30%	dG^+^	232 ± 4	72 ± 2	44 ± 1	<6	<6

All values represent the mean ± deviation of the mean for two analyses

Phages of the first group encoding both a DpdA and one of the amidotransferase homologs were analyzed. *Streptococcus* phage Dp-1 DNA, encoding a QueF-L, contained a large amount of dPreQ_1_ (3389 ± 184 modifications per 10^6^ nucleotides, ~1.7 ± 0.09% of the Gs) but no dG^+^, which would mean that the QueF-L of this phage would actually be functionally closer to bacterial QueF than archaeal QueF-L, as predicted by the SSN clustering (Supplementary Fig. 2). *Vibrio* phage nt-1, encoding an ArcS, was shown to harbor not only dG^+^ (44 ± 1 modifications per 10^6^ nucleotides, ~0.02 ± 0.0005% of the Gs) but also dPreQ_0_ and dADG (232 ± 4 modifications per 10^6^ nucleotides, ~0.11 ± 0.002% of the Gs, and 72 ± 2 modifications per 10^6^ nucleotides, ~0.035 ± 0.001% of the Gs, respectively). This result might indicate that nt-1 DpdA is more promiscuous and could insert all intermediates of the pathway.

Then we investigated phages of the second group that encode both a DpdA and the four proteins of the preQ_0_ biosynthesis pathway but no amidotransferase homolog. *Mycobacterium* phage Rosebush was found to harbor dPreQ_0_ in its DNA (96,530 ± 2529 modifications per 10^6^ nucleotides, ~28 ± 1% of the Gs), as does *Escherichia* phage CAjan (70,628 ± 2445 modifications per 10^6^ nucleotides, ~32 ± 1% of the Gs). However, *Mycobacterium* phage Rosebush *was* also found to harbor a negligible amount of dADG (9 ± 1 modifications per 10^6^ nucleotides, ~0.003 ± 0.0003% of the Gs).

The genomic DNA of *Salmonella* phage 7–11 and *Mycobacterium* phage Orion from the third group of phages, which only encode a DpdA, were also analyzed by LC-MS/MS. *Mycobacterium* phage Orion lacked any 7-deazaguanine modifications in its DNA. This result was expected, as none of the phage nor the host encode for the preQ_0_ biosynthesis pathway (*Mycobacterium smegmatis*, *see*Supplementary Data 3). However, *Salmonella* phage 7–11 was unexpectedly modified by dADG (50 ± 2 modifications per 10^6^ nucleotides, ~0.02 ± 0.0009% of the Gs), suggesting that the phage encoded a protein responsible for the oxidation of preQ_0_.

Finally, Halovirus HVTV-1, which encodes the four proteins of the preQ_0_ biosynthesis pathway and a QueF-L homolog but no DpdA, contained mainly dPreQ_1_ (88,607 ± 3014 modifications per 10^6^ nucleotides, ~30 ± 1% of the Gs) but also relatively small amounts of dADG and dG^+^ (152 ± 3 modifications per 10^6^ nucleotides, ~0.05 ± 0.001% of the Gs, and 22 ± 1 modifications per 10^6^ nucleotides, ~0.008 ± 0.0003% of the Gs, respectively). As its host, *H. vallismortis* harbors a DpdA homolog, and it is possible that the host DpdA inserts preQ_0_ in Halovirus HVTV-1 DNA before it is further modified to dPreQ_1_ or dG^+^ by the viral QueF-L or to dADG by another unidentified protein.

### *dpdA* is essential for DNA modification

To evaluate the role of the 7-deazaguanine modifications in phages, we used the *Escherichia* phage CAjan as a genetic model. CAjan is a virulent phage belonging to the *Seuratvirus* genus of the *Siphoviridae* family with many similarities with *Enterobacteria* phage 9g, particularly within the 7-deazaguanine modification pathway^43^. Using the CRISPR-Cas9 genome editing technology^44^, we generated a CAjan derivative with an inactive allele of the *dpdA* gene (Supplementary Fig. 3A). The presence of this allele was confirmed by PCR and sequencing (Supplementary Fig. 3B). The LC-MS/MS analysis of the DNA of the mutated phage showed a complete lack of 7-deazaguanine modifications (Table 2).

### The DNA modifications protect DNA from restriction enzymes

**Fig. 5:**
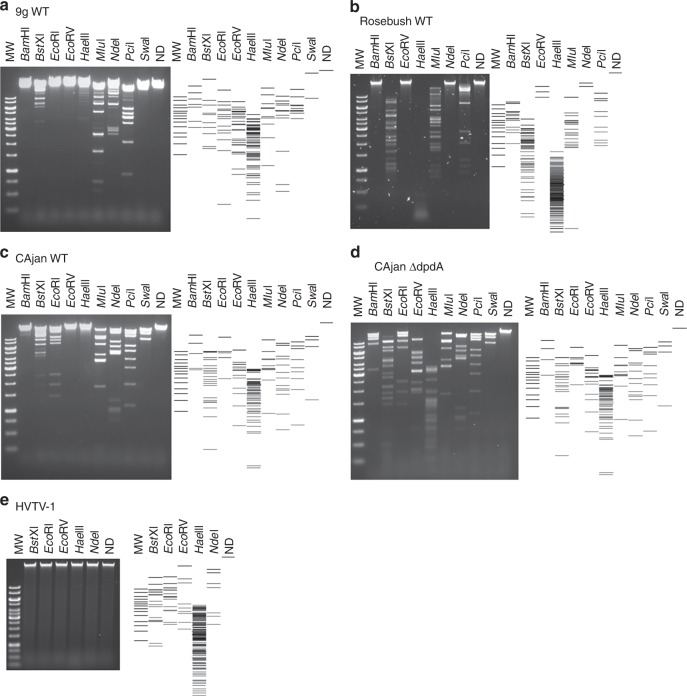
Restriction patterns for phage genomic DNA. Different restriction enzymes were used on the DNA of *Enterobacteria* phage 9g (**a**), *Mycobacterium* phage Rosebush (**b**), *Escherichia* phage CAjan WT (**c**), *Escherichia* phage CAjan Δ*dpdA* (**d**), and Halovirus HVTV-1 (**e**). On the side of each gel is the representation of the expected restriction pattern. Source data are provided as a Source Data file.

The different modifications present in the phages analyzed above may lead to distinct resistance patterns to host defense mechanisms, such as RM systems. To test this hypothesis, phage DNA preparations were digested with a set of restriction enzymes that had been shown to be totally or partially inactivated in the presence of the dG^+^ modification^29^. As a control, we reproduced the results published with *Enterobacteria* phage 9g DNA (Fig. 5a); no digestion was observed with BamHI, EcoRI, EcoRV, and SwaI, while it was partially restricted with BstXI, HaeIII, MluI, NdeI, and PciI.

*Mycobacterium* phage Rosebush DNA that carries preQ_0_ showed a slightly different pattern of resistance. The restriction profiles for BamHI, BstXI, and EcoRV were identical to those of *Enterobacteria* phage 9g. However, Rosebush DNA was fully sensitive to HaeIII, MluI, and PciI and resisted NdeI degradation (Fig. 5b). EcoRI and SwaI could not be tested because the corresponding sites are absent in the *Mycobacterium* phage Rosebush genome.

Though *Escherichia* phage CAjan DNA carries the same modification as *Mycobacterium* phage Rosebush DNA, differences in the restriction patterns were observed (Fig. 5c). Indeed, while EcoRI and SwaI fully digested this DNA preparation, BamHI digested it only partially, and HaeIII did not cut at all. These differences could be explained by the additional small amount of dADG present in *Mycobacterium* phage Rosebush DNA, by the differences in modification density potentially affecting accessibility to the restriction sites, or by the presence of another undetected modification. In comparison, the Δ*dpdA* mutant of CAjan, lacking any modifications, was fully digested by all the tested restriction enzymes (Fig. 5d), formally linking the presence of the *dpdA* gene and the dG^+^ modification to the restriction resistance phenotype.

Last but not least, Halovirus HVTV-1 DNA that carries mainly dPreQ_1_ was found to resist restriction by all enzymes tested, even those that lack guanine in the recognition site (Fig. 5e and Supplementary Fig. 4). It is possible that this virus has other modifications that help resist restriction and, if not dPreQ_1_, is the best modification for protection from restriction enzymes identified in this study.

## Discussion

In a previous study^11^, we identified two 7-deazaguanine modifications in DNA: dADG in bacteria and dG^+^ in phages. Here we added two modifications, dPreQ_1_ and dPreQ_0_, both found in phages. Similar to the result of Szymanski’s group on Campylobacter phages^36^, we also detected dADG in phage genomes. We identified the genes involved in the synthesis of these different modifications. FolE, QueD, and QueE from *Enterobacteria* phage 9g were shown to functionally replace their *E. coli* orthologs (Fig. 2a), and their clustering in SSNs (Supplementary Fig. 2) leaves no doubt on the isofunctionality of these families. No individual phage QueC was tested, but the strong clustering of bacterial, archaeal, and phage QueC proteins in SSNs also point to identical functions. One exception may be the singleton encoded QueC-like protein, found in *Escherichia* phage ECML-4 (YP_009101458 in NC_025446) or *Mycobacterium* phage Muddy (YP_008408902 in NC_022054), which is likely a member of another subfamily of the N-type ATP pyrophosphatases superfamily^38^.

Most 7-deazaguanine-containing phage genomes also harbor a gene coding for a DpdA homolog. As with its bacterial homolog^27^, the phage DpdA introduces PreQ_0_ in DNA (Fig. 2c, Table 1), most likely through a base exchange mechanism similar to its TGT homolog^31^. DpdA2 proteins appear to share this function, as the *Vibrio* phage nt-1 genome contains dPreQ_0_ (Table 2 and Fig. 3). However, not all phages/viruses containing 7-deazaguanines encode DpdA proteins, as observed with Halovirus HVTV-1 (Table 2 and Fig. 3). It is possible that, in the case of HVTV-1, the host DpdA is responsible for the presence of modifications in its genome (EMA11768 in AOLQ01000002). Nevertheless, a DpdA is not always present in the host, and there could be some cases where the phages encode a machinery to synthesize a modified dGTP that is used by DNA polymerase, as proposed for *Campylobacter* phages^36^. Finally, one cannot rule out that some phages may harbor undetected 2′-deoxyribosyltransferases.

**Fig. 6:**
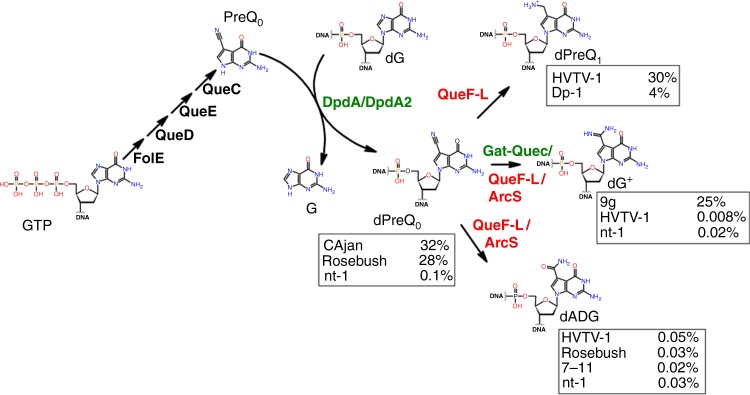
Proposed synthesis pathway for the 2′-deoxy-7-deazaguanine modifications identified in this study. The percentages of modifications identified for each phage are shown in boxes next to the modification of interest. Protein names in green are the reactions identified in this study. In red are the proposed reactions. Molecule abbreviations: guanosine tri-phosphate (GTP), 7-cyano-7-deazaguanine (preQ_0_), 2′-deoxy-7-cyano-7-deazaguanosine (dPreQ_0_), guanine (G), 2′-deoxyguanosine (dG), 2′-deoxy-7-aminomethyl-7-deazaguanosine (dPreQ_1_), 2′-deoxy-7-amido-7-deazaguanosine (dADG), and 2′-deoxyarchaeaosine (dG^+^).

The combination of comparative genomic analyses and experimental validations has allowed pathways for the insertion of dPreQ_0_, dPreQ_1_, and dG^+^ in phage genomes to be predicted (Fig. 6). The presence of the minimal set of FolE, QueD, QueE, QueC, and DpdA proteins leads to the insertion of dPreQ_0,_ as observed in *Mycobacterium* phage Rosebush and *Escherichia* phage CAjan genomes (Table 2 and Fig. 3). The replacement of QueC by Gat-QueC leads to the introduction of dG^+^ (Fig. 2c, Table 1 and previous study^11^). However, it is not known whether Gat-QueC converts preQ_0_ into G^+^ before or after it is inserted into DNA. The function of ArcS homologs in phages/viruses is less clear. Indeed, *Vibrio* phage nt-1 encodes an ArcS homolog, and its DNA contains mainly dPreQ_0_ but also dG^+^ and dADG (Table 2 and Fig. 3). ArcS was the first G^+^ synthase identified in archaea^15^. Based on the phage and archaeal ArcS cluster in the SNNs (Supplementary Fig. 2), it is possible that some phage ArcS protein evolved to perform not only an amidotransferase reaction, such as the archaeal ArcS^15^, but also an amidohydrolase reaction, such as the bacterial DpdC^27^. Further biochemical characterization will be required to explore these hypotheses. One cannot exclude the possibility that the small amount of dADG detected in *Vibrio* phage nt-1, Halovirus HVTV-1, *Mycobacterium* phage Rosebush, and *Escherichia* phage CAjan could be the result of the natural oxidation of dPreQ_0_^45^.

The discrepancy observed between the SSNs and HHpred predictions for the QueF/QueF-L homologs was resolved by analyzing *Streptococcus* phage Dp-1 and Halovirus HVTV-1 DNA. HHpred analysis predicted that a homolog of the archaeal QueF-L, which synthesizes G^+^-tRNA from the preQ_0_-tRNA^46^, was encoded by these phages, whereas the SSN analysis predicted that this same protein was part of a group of bacterial QueF proteins (Fig. 4) that synthesize preQ_1_ from the free preQ_0_ base^18^. We found that *Streptococcus* phage Dp-1 and Halovirus HVTV-1 were modified by dPreQ_1_, confirming the SSN prediction. However, it is unclear whether the reduction occurs on free preQ_0_, similar to the bacterial QueF proteins^18^, and then the free base preQ_1_ is inserted by DpdA or if the phage QueF is able to modify the DNA-bound dPreQ_0_, as does the archaeal QueF-L with tRNA^46^. However, Halovirus HVTV-1 contains mainly dPreQ_1_ but also a small amount of dADG and dG^+^. It is possible that the QueF-L transitions between its function as an amidohydrolase to an amidotransferase, but one cannot rule out that the host ArcS could catalyze the reaction, although the PUA domain specific for tRNA binding makes it highly unlikely^15^.

From a biological perspective, 7-deazaguanine modifications seem to dramatically decrease the susceptibility of phage genomes to host RM systems. RM systems are one of the major defense systems for bacteria to prevent invasion by foreign DNA^5^. Phages evolved to escape these RM systems by different methods, including modification of their genomic DNA^9,11,47,48^. It was previously observed that the genome of *Enterobacteria* phage 9g contains dG^+^^11^ and is fully or partially resistant to a wide variety of restriction enzymes^29^. In this study, we directly linked the presence of the modification to the restriction resistance phenotype. *Escherichia* phage CAjan with mutations in *dpdA* no longer contains dPreQ_0_ modifications (Table 2) and is sensitive to all the restriction enzymes tested (Fig. 5). In addition, all 7-deazaguanine-modified DNA preparations tested were protected to various degrees from digestion by restriction enzymes. We also observed that introducing dG^+^ modifications in the *E. coli* genome protected against cleavage by EcoRI (Fig. 2). These modifications might also block other DNA-binding proteins that require the nitrogen moiety at position 7 of the guanine to recognize their substrates, the most critical being sigma and transcription factors. However, phages only use the housekeeping sigma factor^49^, which has an AT-rich recognition sequence^50^, and encode their own transcription factors^51^.

Finally, the distribution of these modifications among phages seems to correlate with their host range, namely, bacterial pathogenic species. Interestingly, this was also observed in bacteria, where many pathogens harbor dADG modifications^11^. Although it is not clear how 7-deazaguanine modifications are spread through phage isolates, these modifications might give a selective advantage to pathogenic species. These 7-deazaguanine-modified phages are also most likely more adapted to propagate in hosts with modified DNA. We can only speculate on how bacteria evolve to counteract this specific anti-restriction mechanism. As we were successful in deleting the *dpdA* gene from *Escherichia* phage CAjan using a CRISPR-Cas9 technique (see “Methods”), we know that these modifications do not provide resistance against the type II CRISPR-Cas system^4^. However, as the adaptive system of CRISPR-Cas recognizes the nitrogen in position 7 of the guanines in the PAM^52^, it is possible that these phages escape degradation by CRISPR-Cas by preventing the adaptation system from binding to its target DNA. One could also imagine that other means of defense, described in recent reviews^2,3^, provide an efficient protection mechanism against these phages or that some bacteria evolved means of defense yet to be discovered.

## Methods

### Strains, phages, plasmids, and oligonucleotides

The bacterial strains used in this study are listed in Supplementary Data 4. Phages are listed in Supplementary Data 5. Plasmids are listed in Supplementary Table 3, and plasmid constructions are described in Supplementary Information. Oligonucleotides are listed in Supplementary Data 6.

### Q detection in tRNA

Overnight bacterial cultures were diluted 1/100-fold into 5 mL of LB supplemented with 0.4% arabinose and 100 µg/mL ampicillin and grown for 2 h at 37 °C. Cells were harvested by centrifugation at 16,000 × g for 1 min at 4 °C. Cell pellets were immediately resuspended in 1 mL of Trizol (Life Technologies, Carlsbad, CA). Small RNAs were extracted using the PureLink^TM^ miRNA Isolation Kit from Invitrogen (Carlsbad, CA) according to the manufacturer’s protocol. Purified RNAs were eluted in 50 μL of RNase-free water, and tRNA concentrations were measured with a NanoDrop® ND-1000 Spectrophotometer (Thermo Fisher Scientific, Waltham, MA). Then 200 ng of RNA was migrated in a 10% acrylamide/bisacrylamide (29:1), Tris-EDTA acetate (TAE) 1×, Urea 8 M supplemented with 5 µg/mL 3-(acrylamido)-phenylboronic acid, as described in detail previously^27^. The migrated samples were transferred onto a Biodyne^TM^ B Nylon membrane (0.45 µm, Thermo Scientific, Rockford, IL). tRNA samples were detected using a (5′-biotin-CCCTCGGTGACAGGCAGG-3′) probe that anneals with tRNA_Asp_(GUC) at a final concentration of 0.3 μM and the Chemiluminescent Nucleic Acid Detection Module Kit (Thermo Scientific, Rockford, IL), except that the first blocking buffer was changed to the DIG Easy Hyp buffer (Roche, Mannheim, Germany).

### Restriction assay for deazapurine presence in plasmid DNA

*E. coli* strains containing different variations of pBAD24 and pBAD33 (with or without *dpdA* or *gat-queC* from *Enterobacteria* phage 9g, see Supplementary Information) were grown overnight in LB supplemented with ampiciline 100 µg/mL, chloramphenicol 20 µg/mL and 0.2% glucose at 37 °C. Each strain was diluted 100-fold in LB supplemented with ampiciline 100 µg/mL, chloramphenicol 20 µg/mL and﻿ 0.4% arabinose and grown for 6 h at 37 °C. Plasmids were extracted using the Qiagen QIAprep Spin Miniprep Kit, and 500 ng of plasmid was digested by EcoRI-HF (New England Biolabs, Ipswich MA) for 1 h at 37 °C in 20 µL of CutSmart buffer. The enzyme was inactivated by 20-min incubation at 80 °C. The samples were run on a 0.5% agarose gel and TAE 1×. The gel was then stained with 0.5 µg/mL ethidium bromide for 30 min, washed 3 times for 15 min in water, and visualized with the Azur Biosystem c200 Gel Doc system (Thermo Fisher Scientific, Waltham, MA, USA).

### Search for phage encoding Q and G^+^ biosynthesis proteins

The Viruses nr database from NCBI was queried by three iterations of PSI-BLAST^53^, with the default set-up as previously suggested^54^, using the proteins referenced in Supplementary Table 1 known to be involved in Q or G^+^ biosynthesis, as well as DpdA from *Enterobacteria* phage 9g, predicted to be involved in the modification of phage DNA, and another DpdA2 from *Vibrio* phage nt-1, part of a family identified in this study. The preQ_0_-specific transporter YhhQ^33^ was also added. For each virus identified with at least one of these genes, a reverse analysis was performed (phage genome against the protein list) to ensure that no protein was missed during the first analysis. The annotations for each identified ortholog were verified by HHpred^55^.

### SSN generation

For each protein family (FolE, QueD, QueE, QueC/Gat-QueC, QueF/QueF-L, ArcS, and TGT), a representative set was imported from the OMA database^56^. For the DpdA from bacteria, the protein sequences were imported from the genomes identified previously^11^ through PubSEED^42^. To generate the protein network, the sequences in fasta format were uploaded and analyzed online by the EFI-EST tool^37^. Each network was analyzed using the Cytoscape program^57^, and each family was clustered using the alignment score thresholds indicated in Fig. 3 and Supplementary Fig. 2.

### Identification of the host and their gene content

The Virus-Host DB^41^ was used to obtain the host information for each phage identified in this study. For phages not referenced in this database, a manual investigation coupling RefSeq^39^ and the literature was performed (indicated as “manual” in the evidence line of Supplementary Data 3). Each host identified was queried in the Globi database^40^, and if they were identified as pathogens, the host was entered in the “Pathogen Of” column of Supplementary Data 3. The same analysis was performed for all the dsDNA phages of the Virus-Host DB, as only these phages were returned in our analysis (data not shown). A list of genomes was created on PubSEED^42^ from the identified hosts, and a spreadsheet was created. Proteins from Supplementary Table 1 were used to identify the correct annotation for each column of the spreadsheet. The results were collected and are shown in Supplementary Data 3.

### Purification of phage and plasmid DNA

The purification of each phage DNA in this study was performed specifically for each phage and is described in Supplementary Information.

### Mass spectrometric analysis

DNA analysis was performed as previously described with several modifications^11^. Purified DNA (20 μg) was hydrolyzed in 10 mM Tris-HCl (pH 7.9) with 1 mM MgCl_2_ with benzonase (20 U), DNase I (4 U), calf intestine phosphatase (17 U), and phosphodiesterase (0.2 U) for 16 h at ambient temperature. Following passage through a 10-kDa filter to remove proteins, the filtrate was lyophilized and resuspended to a final concentration of 0.2 µg/µL (based on initial DNA quantity).

Quantification of the modified 2′-deoxynucleosides (dADG, dQ, dPreQ_0_, dPreQ_1_, and dG^+^) and the four canonical 2′-deoxyribonucleosides (dA, dT, dG, and dC) was achieved by LC-MS/MS and an in-line diode array detector (LC-DAD), respectively. Aliquots of hydrolyzed DNA were injected onto a Phenomenex Luna Omega Polar C18 column (2.1 × 100 mm, 1.6 μm particle size) equilibrated with 98% solvent A (0.1% v/v formic acid in water) and 2% solvent B (0.1% v/v formic acid in acetonitrile) at a flow rate of 0.25 mL/min and eluted with the following solvent gradient: 12% B for 10 min, 1 min ramp to 100% B for 10 min, 1 min ramp to 2% B for 10 min. The high-performance liquid chromatographic column was coupled to an Agilent 1290 Infinity DAD and an Agilent 6490 triple quadruple mass spectrometer (Agilent, Santa Clara, CA). The column was kept at 40 °C, and the autosampler was cooled at 4 °C. The ultraviolet wavelength of the DAD was set at 260 nm and the electrospray ionization of the mass spectrometer was performed in positive ion mode with the following source parameters: drying gas temperature, 200 °C with a flow of 14 L/min; nebulizer gas pressure, 30 psi; sheath gas temperature, 400 °C with a flow of 11 L/min; capillary voltage, 3,000 V; and nozzle voltage, 800 V. Compounds were quantified in multiple reaction monitoring mode with the following *m*/*z* transitions: 310.1 → 194.1, 310.1 → 177.1, 310.1 → 293.1 for dADG; 394.1 → 163.1, 394.1 → 146.1, 394.1 → 121.1 for dQ; 292.1 → 176.1, 176.1 → 159.1, 176.1 → 52.1 for dPreQ; 296.1 → 163.1, 296.1 → 121.1, 296.1 → 279.1 for dPreQ_1_; and 309.1 → 193.1, 309.1 → 176.1, 309.1 → 159.1 for dG^+^. External calibration curves were used to quantify the modified canonical 2′-deoxynucleosides. Calibration curves were constructed from replicate measurements of eight concentrations of each standard. A linear regression with *r*^2^ > 0.995 was obtained in all relevant ranges. The limit of detection, defined by a signal-to-noise ratio ≥3, ranged from 0.1 to 1 fmol for the modified 2′-deoxynucleosides. Data acquisition and processing were performed using the MassHunter software (Agilent, Santa Clara, CA).

### Phage genome editing using CRISPR-Cas9

*Escherichia* phage CAjan was genetically engineered as previously described^58^ and as summarized in Supplementary Fig. 2A. Briefly, *E. coli* MG1655 was transformed with two plasmids, pL2Cas9_dpdAΔ (see Supplementary Information for detailed construction method), which contained a spacer (5′-TGCGGTCAAGCCAAGTCTTAAGCGTGTCCG-3′) targeting the *dpdA* gene of *Escherichia* phage CAjan, and pNZ123_dpdAΔ (see Supplementary Information for detailed construction method), which carried a homologous repair template with a partially deleted, nonfunctional allele of the *dpdA* gene (del29212-29521). Phage engineering was accomplished by infecting the modified host with WT *Escherichia* phage CAjan and isolating the resulting phage mutants. The infection step was repeated twice, and the resulting mutants were verified by PCR and whole-genome sequencing as described elsewhere^59^.

### Restriction assay of phage DNA

A total of 250 ng of phage DNA was digested by the enzymes (New England Biolabs) described in Fig. 6 for 1 h at 37 °C in 20 µL of CutSmart or 3.1 Buffer solution, according to the manufacturer’s instructions. The enzymes were inactivated by incubation at 80 °C for 20 min. The samples were run on a 0.7% agarose gel and TAE 1×. The gel was then stained for 30 min in 0.5 μg/mL ethidium bromide, washed 3 times for 15 min in water, and visualized with the Azur Biosystem c200 Gel Doc system.

### Reporting summary

Further information on research design is available in the Nature Research Reporting Summary linked to this article.

## Supplementary information

Supplementary Information

Peer Review

Reporting Summary

Description of Additional Supplementary Files

Suplementary data 1

Suplementary data 2

Suplementary data 3

Suplementary data 4

Suplementary data 5

Suplementary data 6

## Data Availability

Data supporting the findings of this work are available within the paper and its Supplementary Information files. A reporting summary for this article is available as a Supplementary Information file. The datasets generated and analyzed during the current study are available in Supplementary Information or from the corresponding author upon request. The source data are provided as a Source Data file.

## Source data

Source Data
