# Supplementary material for: 7-Deazaguanine modifications protect phage DNA from host restriction systems
Source: Nat Commun. 2019 Nov 29;10:5442. doi: 10.1038/s41467-019-13384-y (PMC6884629; doi:10.1038/s41467-019-13384-y)
Supplement: Supplementary file 1 — Supplementary Information [file 41467_2019_13384_MOESM1_ESM.pdf]

7-Deazaguanine modifications protect phage DNA from host restriction systems. – Supplementary material

Geoffrey Hutinet[1,*], Witold Kot[2], Liang Cui[3], Roman Hillebrand[4†], Seetharamsingh Balamkundu[3], Shanmugavel Gnanakalai[3], Ramesh Neelakandan[3], Alexander B. Carstens[2], Chuan Fa Lui[5], Denise Tremblay[6,7], Deborah Jacobs-Sera[8], Mandana Sassanfar[9], Yan-Jiun Lee[10], Peter Weigele[10], Sylvain Moineau[6,7], Graham F. Hatfull[8], Peter C. Dedon[3,4], Lars H. Hansen[2] and Valérie de Crécy-Lagard[1,11,*]

[1] Department of Microbiology and Cell Science, University of Florida, Gainesville, FL 32611

[2] Department of Environmental Science, Aarhus University, Roskilde, Denmark

[3] Singapore-MIT Alliance for Research and Technology, Antimicrobial Resistance Interdisciplinary Research Group, Campus for Research Excellence and Technological Enterprise, Singapore 138602, Singapore

[4] Department of Biological Engineering and Center for Environmental Health Sciences, Massachusetts Institute of Technology, Cambridge, MA 02139

[5] School of Biological Sciences, Nanyang Technological University, 60 Nanyang Drive Singapore 637551

[6] Département de biochimie, microbiologie et de bio-informatique, Faculté des sciences et de génie, Université Laval, Québec City, QC, Canada, G1V 0A6

[7] Félix d'Hérelle Reference Center for Bacterial Viruses and Groupe de recherche en écologie buccale, Faculté de médecine dentaire, Université Laval, Québec City, QC, Canada, G1V 0A6

[8] Pittsburgh Bacteriophage Institute and Department of Biological Sciences, University of Pittsburgh, Pittsburgh, PA 15260

[9] Department of Biology, Massachusetts Institute of Technology, Cambridge MA 02139

[10] Research Department, New England Biolabs, Ipswich, MA 01938

[11] University of Florida, Genetics Institute, Gainesville, Florida 32610

[†] Present address: Nitto Denko Avecia, 125 Fortune Boulevard, Milford MA 01757

* Corresponding authors: ghutinet@ufl.edu and vcrecy@ufl.edu

Supplementary Methods

Media composition

Lysogeny broth[1] (LB): 10 g/L tryptone, 5 g/L yeast extract, 10 g/L NaCl, powder order from Thermo Fisher Scientofic (BP1426).

Brain heart infusion[2] (BHI): Merck cat. 110493

BHI+[3]: BHI supplemented with 8 µM $MnCl_2$, 0.25 mM, $CaCl_2$, 0.2 mM $MgSO_4$, 50 mM Tris-HCl pH 7.5, 50 ng/µl choline chloride, 0.4% glycine and 100 µl/ml catalase.

Middlebrook 7H9 broth: 4.7 g Middlebrook 7H9 (Difco), 5 mL 40% glycerol, 900 mL ddH2O.

Middlebrook 7H10 agar: 19.0 g Middlebrook 7H10 (Difco), 12.5 mL 40% glycerol, 4.95 mL 40% dextrose, 5 drops anti-bubble, 990 mL ddH2O.

Middlebrook Top Agar: 4.7g Middlebrook 7H9 (Difco), 7.0 g BactoAgar, ddH2O up to 1000 mL, 4 drops of anti-bubble.

Salt water (SW) stock (30%): 240 g/L NaCl, 30 g/L $MgCl_2$, 35 g/L $MgSO_4$, 7 g/L KCl, 5 mM Tris-HCl pH 7.5.

Modified growth medium (Rodrigez-Valera 1983) (MGM): for liquid broth 23 % SW is used, 20 % for agar medium and 18 % for soft-agar medium. 5 g/L peptone and 1 g/L yeast extract are also added.

Difco nutrient broth: 3 g/L beef extract, 5 g/L peptone.

To these media, 15 g/L of agar are uses for solid medium and 7 g/L for top-agar medium.

Construction of the *E. coli* Q^- mutants

The *E. coli* BW25113 *folE::kan*, *queD::kan*, *queE::kan*, *queC::kan* and *tgt::kan* mutants were collected from the Keio collection[4]. Each mutation was transduced using phage P1[5] in MG1655. The transductions were verified by PCR (couple of primers used: GO119/GO120 and GO121/GO122 for *folE* mutation, GO123/GO124 and GO125/GO126 for *queD* mutation, GO127/GO128 and GO129/GO130 for *queE* mutation, GO111/GO112 and GO113/GO114 for *queC* mutation, GO107/GO108 and GO109/GO110 for *tgt* mutation). The kanamycin cassette was removed from all these strains but Δ*tgt* using pCP20 as described by Datsenko and Wanner[6]. The resulting strains are listed in **Supplementary Data 4**.

Cloning of *E. coli tgt*

The *tgt* gene was amplified by PCR from *E. coli* MG1655 using primers tgt_pBAD24_KpnI_F and tgt_pBAD24_SphI_R. The resulting PCR product and pBAD24 were digested by KpnI and SphI (NEB), following the recommendation of the manufacturer. The PCR product and the plasmid were then ligated using the T4 DNA ligase from NEB, following the manufacturer recommendations. The resulting plasmid was verified by sequencing and is listed in **Supplementary Table 3**.

Cloning of *Enterobacteria* phage 9g genes

dpdA, *folE*, *queD*, *queE* and *gat-queC* genes from *Enterobacteria* phage 9g (see **Supplementary Data 1** for accession numbers) were amplified by PCR using the following pairs of primers: GO80/GO81, GO92/GO93, GO94/GO95, GO100/GO101 and GO96/GO97, respectively. pBAD24 plasmid and the PCR products were digested by SalI-HF and SbfI-HF (NEB). The genes were then inserted by ligation using the T4 DNA

ligase from NEB. *dpdA* and *gat-queC* were also cloned in pBAD33 using the same methods. The resulting plasmids were verified by sequencing and are listed in **Supplementary Table 3**. Each resulting plasmid was transformed in different mutants of *E. coli* MG1655 as listed in **Supplementary Data 4** for the experiment showed in **Figure 2A**. Different pairs of plasmids were co-transformed in *E. coli* MG1655, *E. coli* MG1655 Δ*queC*, *E. coli* MG1655 Δ*queD* or *E. coli* MG1655 Δ*tgt* as listed in **Supplementary Data 4** for the experiment showed in **Figures 2BC**.

Isolation and characterization of *Pseudomonas* phage Quinobequin P09

A phage infecting *Pseudomonas aeruginosa* strain UCBPP-PA14[7] was isolated from a Charles River water sample collected in the Summer of 2015 using enrichment methods[8] and plaque purification. This phage was renamed "Quinobequin" after the indigenous name of the river from whence it came. Quinobequin P09 was propagated at two liter scale, purified, and virion genomic DNA extracted as described previously[9]. Genomic DNA was sequenced using Pacific Biosciences SMRT sequencing technology[10] and the resulting 58,277 bp circularly permuted dsDNA genome was annotated using the RAST annotation pipeline[11].

Quinobequin P09 shares many genomic features with the *Enterobacteria* phage 9g (NC_024146) and the *Pseudomonas* phage PaMx25 (JQ067084) including highly homologous and syntenic structural genes (indicating a lambda-like *siphovirus* morphology) and biosynthetic gene cluster encoding a suite of enzymes for the synthesis and installation of a 7-deazaguanine derivative nucleotide (**Supplementary Figure 5A**). High performance liquid chromatography (HPLC) separation and mass spectrometry (MS) analysis of nucleosides released by enzymatic hydrolysis of Quinobequin P09 virion DNA using previously described methods[9] revealed the presence of a fifth nucleoside species having a mass of 308 Daltons (**Supplementary Figure 5B**), matching the mass of dG^+, previously shown to occur in the genomic DNA of phage 9g[12]. Integration of peak areas for canonical nucleotides detected by UV absorbance show a ratio of G to C less than one, indicating the 308 Da nucleoside partially substitutes for G. Subsequent analysis by collision induced dissociation (CID) during MS revealed a fragmentation pattern consistent with the structure of dG^+ as previously reported[12]. The electron microscopy of *Pseudomonas* phages Quinobequin P09 confirmed the *siphovirus* morphology (**Supplementary Figure 5C**).

Plasmid DNA preparation for mass spectrometry

Overnight cultures were diluted 1/100-fold into 500 mL of LB supplemented with 0.4 % arabinose, 100 µg/mL ampicillin and 20 µg/mL of chloramphenicol. Cells were grown overnight and pelleted. The Qiagen maxi-prep kit was used to extract the plasmid following the recommendations of the manufacturer.

CAjan, Dp-1, nt-1 and 7-11 DNA purification

One liter of early log-phase host bacterial culture was equally divided into two, sterile 1 L bottles. Thereafter, one of the bottles was infected with the corresponding phage at a low multiplicity of infection (~ 0.01). All bottles were incubated in the corresponding temperatures and the phage-infected cultures were fed with 250 mL of uninfected host culture after 4 and 6 h post initial infection. Afterwards bottles were incubated overnight

for cell lysis to occur. The next day, cellular debris were removed by centrifugation at 6,000 × g for 10 min. Phage particles were precipitated from the supernatant with 10 % PEG8000 at 4 °C overnight then harvested by centrifugation at 11,000 × g. Supernatant was carefully removed, and the phage pellet was resuspended in 8 mL of SM buffer (100 mM sodium chloride, 10 mM magnesium sulfate, 50 mM Tris, pH 7.5, and 0.01 % gelatin) then filtered through a 0.45 µm syringe filter. Then, 300 µL of the filtered phage solution was treated with 5 µL of DNase I (1 mg/µL) at 37 °C for 30 min. SDS and proteinase K were next added to a final concentration of 0.5 % and 50 µg/mL, respectively, to the samples which were then incubated at 55°C for 1 h. Next, 200 µL of 7.5 M NH_3Ac followed by 500 µL of phenol:chloroform (50:50) mix were added. The mixture was thoroughly vortexed and centrifuged at 18,000 x g for 30 min. The aqueous phase was transferred to a new tube and the DNA was ethanol precipitated. The DNA pellet was dissolved in 50 µL of TE (10mM Tris-HCl, 1mM EDTA) buffer pH 7.5.

Rosebush and Orion DNA purification

Mycobacteria phage Rosebush and Orion were grown as described previously[13]. In brief, 30 mL of a dense *M. smegmatis* culture was mixed with approximately 10^6 phage particles, 270 mL of top-agar were added and the mixture was plated on 30 large (150 x 10mm) solid media plates. After incubation for 36-48 h at 37 °C, 10 mL of phage buffer were added, incubated for 4 h at room temperature, and the phage lysate collected. Following clarification by centrifugation, phage particles were precipitated with the addition of NaCl to a final concentration of 1 M and polyethylene glycol 8000 to a final concentration of 10 %. The precipitated phage particles were collected by centrifugation for 10 minutes at 5,500 x g at 4 °C, and resuspended in 10 mL of phage buffer. The lysate was clarified by centrifuged at 5,500 x g for 10 minutes at 4 °C, 8.5 g of CsCl was added, and placed in a heat-sealed tube. Samples were centrifuged at 38,000 RPM (98,000 x g) for 16 hours, and the visible phage band removed with a syringe through the side of the tube.

Prior to DNA extraction, CsCl was removed by dialysis against phage buffer overnight at 4 °C. For DNA extraction, 0.5 mL of phage lysate (~ 10^{12} particles) were incubated with 12.5 mM $MgCl_2$, 0.8 µU/mL DNAse I and 100 µg/mL RNAse at room temperature for 30 minutes. To this, 20 mM EDTA, 50 µg/mL of Proteinase K and 0.5 % of SDS were added, vortexed vigorously and incubated at 55 °C for 60 minutes. An equal volume of phenol:chlorophorm:isoamyl-alcohol (25:24:1) was added and the mixture was inverted several time before being centrifuged for 5 minutes at room temperature at 13,000 rpm (16,000 x g). This step was repeated several times on the aqueous phase obtained until the white interphase was gone. DNA was ethanol precipitated from the sample, pelleted, washed with 500 µL of 70 % ethanol, dried, and resuspended in 50 µL ddH2O. Concentration was measured using a NanoDrop® ND-1000 Spectrophotometer.

HVTV-1 DNA purification

To 30 mL of a stationary phase *Haloarcula Valismoris* grown in MGM 23 %, phages were added to obtain confluent lysis on plates. Then, 270 mL of MGM 18 % top-agar were added and the mixture was plated on MGM 20 % agar. Phages were grown for 4-5 days at 37 °C then a top layer of HVTV-1 virus buffer[14] (1.2 M NaCl, 44 mM $MgCl_2$, 47 mM $MgSO_4$, 1.5 mM $CaCl_2$, 28 mM KCl, 24 mM Tris-HCl pH 7.2) was poured on top of

each plate. Phages were allowed to diffuse to the liquid phase for 4 h at 4 °C before being harvested. Debris were pelleted and phages were precipitated over night at 4 °C by adding 10 % polyethylene glycol (PEG 8000©) to the supernatant. The phage suspension was centrifuged for 10 minutes at 4,500 x g at 4 °C. The phage pellet was resuspended in 10 mL of HVTV-1 virus buffer and dialyzed in the same buffer over night at 4 °C to eliminate the last traces of PEG. Then, 12.5 mM $MgCl_2$, 0.8 µU/mL DNAse I and 100 µg/mL RNAse were added and the mixture were incubated at room temperature for ~ 30 minutes. Next, 20 mM EDTA, 50 µg/mL of Proteinase K and 0.5 % of SDS were added to the mixture, then vortexed vigorously and incubated at 55 °C for 60 minutes. A equal volume of phenol:chlorophorm:isoamyl-alcohol (25:24:1) was added and the mixture was inverted several time before being centrifuged for 5 minutes at room temperature at 4,500 x g. This step was repeated several times on the aqueous phase obtained until the white interphase was gone. An equal volume of chloroform was added to the aqueous phase, vortexed and centrifuged again to eliminate the last traces of phenol. The DNA was then ethanol precipitated from the sample and pelleted. The pellet was washed with 500 µL of 70 % ethanol. The dried DNA pellet was then resuspended in ~ 50 µL dH_2O. Concentration was measured using a NanoDrop® ND-1000 Spectrophotometer (Thermo scientific, Waltham, MA).

9g DNA purification

To 30 mL of a stationary phase *E. coli* MG1655 grown in LB, phages were added to obtain confluent lysis on plates. Then, 270 mL of LB top-agar were added and the mixture was completely plated on LB agar. The phages were replicated overnight at 37 °C then a top layer of TM buffer (10 mM $MgSO_4$, 10 mM Tris-HCl pH 7.5) was poured on top of each plate. Phages were allowed to diffuse to the liquid phase for 4 h at 4 °C before being harvested. Debris were pelleted, and phages were precipitated over night at 4 °C by adding 1 M of NaCl and 10 % polyethylene glycol (PEG 8000©) to the supernatant. The phage suspension was centrifuged for 10 minutes at 4,500 x g at 4 °C. The phage pellet was resuspended in 10 mL of TM buffer and dialyzed in the same buffer over night at 4 °C to eliminate the last traces of PEG. Next, 12.5 mM $MgCl_2$, 0.8 µU/mL DNAse I and 100 µg/mL RNAse were added and the mixture were incubated at room temperature for ~ 30 minutes. Then, 20 mM EDTA, 50 µg/mL of Proteinase K and 0.5 % of SDS were added to the mixture, which was vortexed vigorously and incubate at 55 °C for 60 minutes. A equal volume of phenol:chlorophorm:isoamyl-alcohol (25:24:1) was then added and the mixture was inverted several time before being centrifuged for 5 minutes at room temperature at 4,500 x g. This step was repeated several times on the aqueous phase obtained until the white interphase was gone. An equal volume of chloroform was added to the aqueous phase, vortexed and centrifuged again to eliminate the last traces of phenol. The DNA was then ethanol precipitated from the sample and pelleted. The pellet was washed with 500 µL of 70 % ethanol. The dried DNA pellet was then resuspended in ~ 50 µL dH_2O. Concentrations were measured using a NanoDrop® ND-1000 Spectrophotometer (Thermo scientific, Waltham, MA).

pL2Cas9_dpdAΔ construction

Oligos containing spacer were phosphorylated. Briefly, 2 µl of DpdA_1 oligo (50 µM) and 2 µl of DpdA_2 oligo (50 µM) were mixed with 10 µL of 5x T4 DNA Ligase buffer

(Life Technologies), 1 µL of T4 Kinase (Life Technologies) and 32 µL of ddH_2O and incubated for 30 minutes at 37 °C followed by 20 minutes at 65 °C. Afterwards, 2 µg of pL2Cas9 plasmid was digested with BsaI enzyme (NEB). Next, the phosphorylated oligos were ligated with to the linearized pL2Cas9 (molar ratio 5:1) using T4 DNA ligase (Life technologies). The next day the ligation product was microdialyzed on a membrane and electroporated in *E. coli* MG1655.

pNZ123_dpdAΔ construction

Repair template was prepared using method proposed by Gibson et al.[15]. Firstly, two PCR products of *dpdA* gene from CAjan were obtained using primers pairs Dpd_L_F with Dpd_L_R and Dpd_R_F with Dpd_R_R and Phusion polymerase (Thermo Fisher Scientific) following manufacturer's protocol. Products were purified on a Clean-up column (A&A Biotechnology). Two micrograms of pNZ123 was digested with *Xba*I enzyme (NEB). Afterwards assembly was performed as One-step isothermal DNA assembly by Gibson et al.[15] using 200 ng of plasmid and 50 ng of each PCR product. The assembled product was microdialyzed on a membrane and electroporated in *E. coli* MG1655/pL2Cas9_dpdAΔ.

Synthesis of 2-Amino-7-(2-deoxy-β-D-erythro-pentofuranosyl)-4,7-dihydro-4-oxo-1H-pyrrolo[2,3- d]pyrimidine-5-carboxamide (dADG)

To a solution of compound S1[16] (130 mg, 0.33 mmol, **Supplementary Figure 6A**) in 1:1 MeOH-dioxane (12 mL) was added Et_3N (0.2 mL, 1.5 mmol) and purged with CO gas for 10 min followed by addition of $Pd(PhCN)_2Cl_2$ (12.7 mg, 0.03 mmol). The reaction mixture was stirred at 60 °C for 24 h, cooled to ambient temperature and evaporated. To the resulting crude ester (S2, **Supplementary Figure 6A**) was added aqueous ammonia (15 mL) in a sealed tube, which was heated at 100 °C for 1 h. The reaction mixture was cooled to ambient temperature and evaporated to dryness. The crude reaction mixture was washed with hot methanol to afford dADG (60 mg, 58 %) as off-white solid. HRMS (ESI): m/z calculated for $C_{12}H_{16}N_5O_5$ $[M+H]^+$ 310.1151, observed 310.1152.

Synthesis of 2-amino-7-(2-deoxy-β-D-erythro-pentofuranosyl)-4,7-dihydro-4-oxo-3H-pyrrolo[2,3- d]pyrimidine-5-carbonitrile (dPreQ$_0$)[17].

To a suspension of S1[16] (600 mg, 1.53 mmol, **Supplementary Figure 6A**) in pyridine (10 mL) was added CuCN (1.37 g, 15.3 mmol) with stirring under reflux for 20 h. The reaction mixture was cooled to ambient temperature and solvent evaporated. The resulting solid was washed thoroughly with 20 % MeOH in dichloromethane, with the washings combined, evaporated and purified by column chromatography (100-200 mesh silica gel) eluting with 10 % to 20 % MeOH in dichloromethane to afford dPreQ$_0$ (220 mg, 49 %) as off-white solid. HRMS (ESI): m/z calculated for $C_{12}H_{14}N_5O_4$ $[M+H]^+$ 292.1046, observed 292.1043.

Synthesis of 2-Amino-7-(2-deoxy-β-D-erythro-pentofuranosyl)-4,7-dihydro-4-oxo-3H-pyrrolo[2,3- d]pyrimidine-5-carboximidamide (dG$^+$)

Dry HCl gas was bubbled through a suspension of dPreQ$_0$ (100 mg, 0.34 mmol) in anhydrous MeOH (20 mL) at 0 °C for 2 h. Following stirring at ambient temperature for

16 h, the reaction mixture was evaporated and treated with 7N NH_3 in MeOH at 0 °C, with stirring for 16 h. The crude reaction mixture was evaporated under vacuum and purified by MPLC using C18 column eluting with acetonitrile and H_2O. The fractions containing product was lyophilized to afford dG^+ (20 mg, 18 %) as an off-white solid[18]. HRMS (ESI): m/z calculated for $C_{12}H_{17}N_6O_4$ $[M+H]^+$ 309.1311, observed 309.1306.

Synthesis of 2-Amino-5-(aminomethyl)-7-(2-deoxy-β-D-erythro-pentofuranosyl)-3,7-dihydro-4-*H*-Pyrrolo[2,3-d] pyrimidin-4-one (dPreQ$_1$)

dPreQ$_1$ was synthesized using procedures as previously reported[19] and purified by preparative HPLC using C18 column and eluting with buffer A H_2O (containing 0.045 % TFA) and buffer B acetonitrile (containing 0.045 % TFA). HRMS (ESI): *m/z* calculated for $C_{12}H_{18}N_5O_4$ $[M+H]^+$ 296.1359, observed 296.1345.

Synthesis of 2-Amino-5-((((1*R*,4*R*,5*S*)-4,5-dihydroxycyclopent-2-en-1-yl)amino)methyl)-7-((2*R*,4*S*,5*R*)-4-hydroxy-5-(hydroxymethyl)tetrahydrofuran-2-yl)-3,7-dihydro-4H-pyrrolo[2,3-d]pyrimidin-4-one (dQ)

Di-*tert*-butyl decarbonate (Boc_2O) and 4-dimethylaminopyridine (DMAP) were added to a solution of compound S3[20] (**Suplementary Figure 6B**) in acetonitrile and stirred at ambient temperature for 48 h. Following solvent reduction under vacuum, the reaction products were purified by column chromatography to afford the Boc-protected intermediate which on treatment with sodium methoxide (NaOMe) in tetrahydrofuan (THF) for 10 min afforded compound S4 (**Suplementary Figure 6B**). Compound S4 and Hoffer's chloro sugar were coupled to give S5 using similar procedures reported by Wang *et al*.[19]. The tolulyl protecting groups were removed with $Mg(OMe)_2$ in MeOH, acetonide and Boc protecting groups were removed using 4 N HCl in dioxane. The crude dQ was purified by preparative HPLC using a C18 column and eluting with buffer A (aqueous 0.045 % TFA) and buffer B (acetonitrile containing 0.045 % TFA). High-resolution mass spectrometry (ESI): *m/z* calculated for $C_{17}H_{24}N_5O_6$ $[M+H]^+$ 394.1727, observed 394.1709.

Supplementary Figures

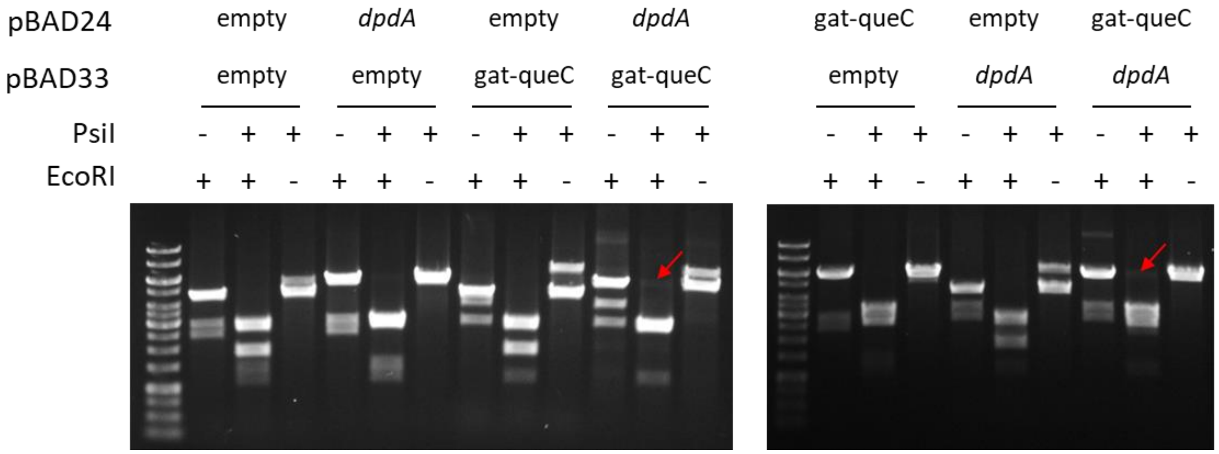

Supplementary Figure 1: Single and double digestion of pBAD24 and pBAD33 derivative plasmids. Plasmids extracted from a *E. coli* WT strains after induction of the plasmids by 0.2 % arabinose. The pBAD24 and pBAD33 line indicate the gene from *Enterobacteria* phage 9g cloned into these plasmids. The PsiI and EcoRI line indicate the presence (+) or absence (-) of these enzymes in the digestion reactions, done 1 h at 37 °C in the buffer recommended by the manufacturer (NEB). The red arrows point at PsiI-linearized plasmid undigested by EcoRI.

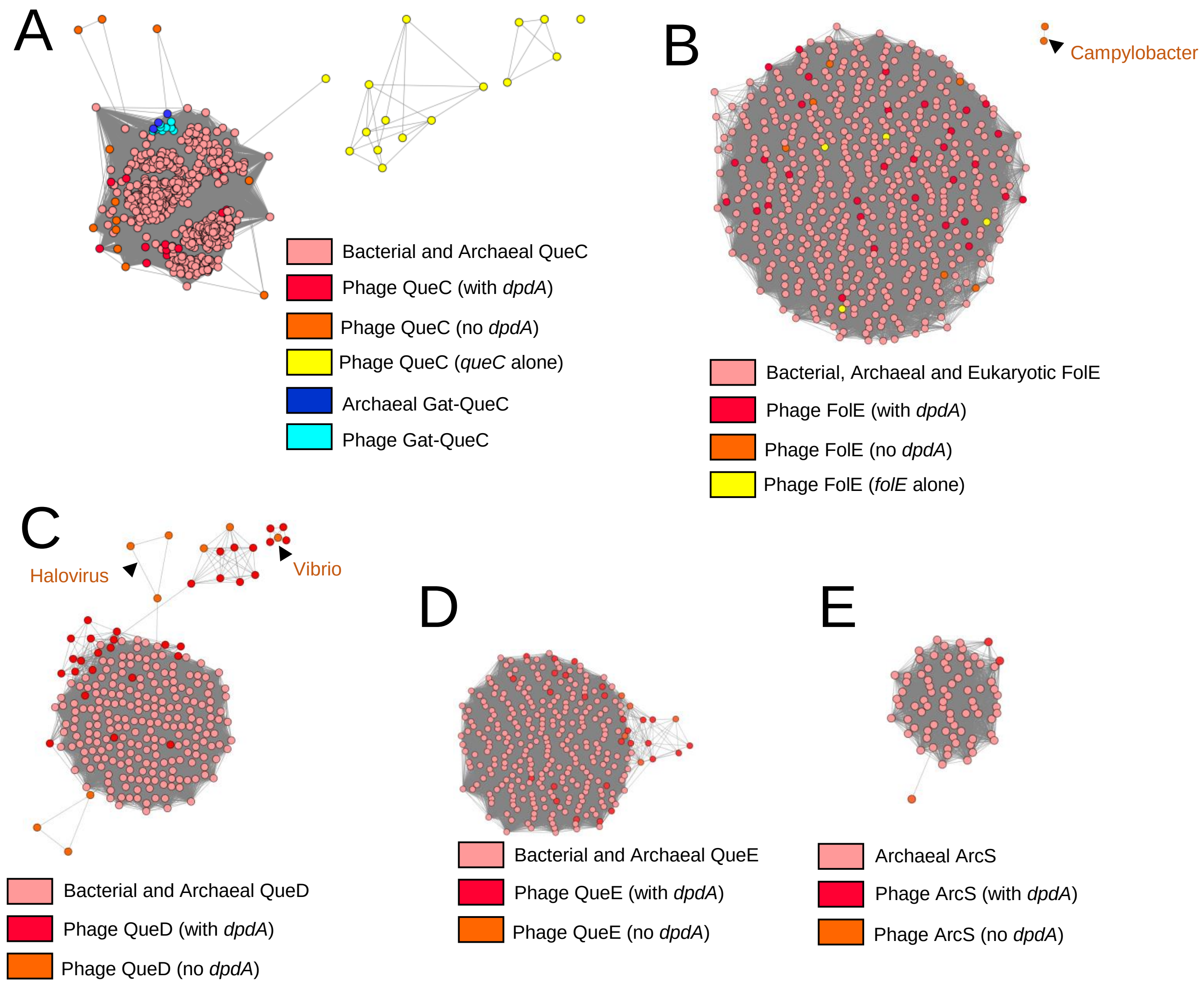

Supplementary Figure 2: Protein Sequence Similarity Network A. QueC protein network, with a threshold alignment score of 10. In light red, the QueC from bacteria; in dark red, the QueC from phages that encode a DpdA; in orange, the QueC from phages that are not encoding a DpdA; and in yellow, the QueC from phage encoding only a QueC. Gat-QueC from archaea is in dark blue and from phages is in light blue. **B.** FolE protein network, each node is a group of proteins identical at 90 %, each edge presents an alignment score above 30. The FolE from archaea, bacteria and eukaryotes are shown in light red. The FolE of phage identified are separated depending on the gene content of phages: in red, FolE in genomes encoding DpdA, in orange the genomes without *dpdA*, in yellow, the genomes with only *folE*. **B.** QueD protein network, with an alignment score threshold of 10. In light red, the archaeal and baterial QueD, in dark red the QueD from phage encoding DpdA, in orange the QueD from phages not encoding DpdA. **C.** QueE protein network, with an alignment score threshold of 13. In light red, the archaeal and baterial QueE, in dark red the QueE from phage encoding DpdA, in orange the QueE from phages not encoding DpdA. **D.** ArcS protein network, with an alignment score threshold of 10. In light red, the archaeal ArcS, in dark red the ArcS from phage encoding DpdA, in orange the ArcS from phages not encoding DpdA.

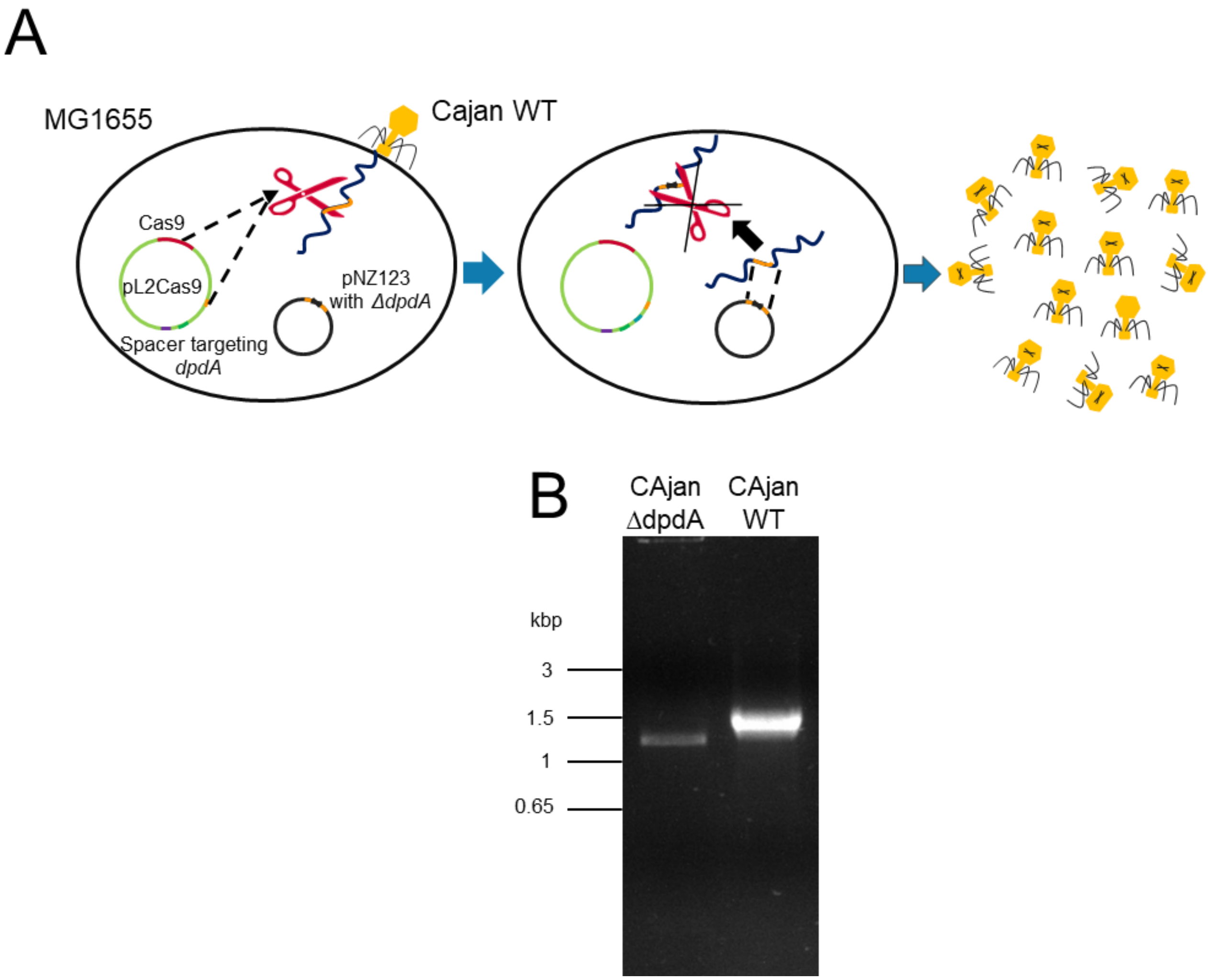

Supplementary Figure 3: Escherichia phage CAjan mutagenesis. A. Targeted genome editing of phage CAjan using CRISPR-Cas9 technology. Host (*E. coli* MG1655) containing the plasmids pNZ123_dpdAΔ and pL2Cas9_dpdAΔ was infected with the wild-type *Escherichia* phage CAjan phage. When the viral DNA enters the cell, the CRISPR-Cas9 system on pL2Cas9_dpdAΔ cleaves the targeted protospacer in *Escherichia* phage CAjan genome. The break is repaired by homologous recombination using the recombination template on pNZ123_dpdAΔ with an allele of *dpdA* carrying a deletion. The recombined genome lacks the protospacer, therefore avoiding cleavage by th CRISPR-Cas9 system. As such, genome edited phages are being released following the completion of the phage lytic cycle. **B.** PCR verification of *Escherichia* phage CAjan *dpdA* mutant. The primers Dpd_F and Dpd_R (**Supplementary Data 6**) were used on genomic DNA from *Escherichia* phage CAjan dpdAΔ (lane 1) and *Escherichia* phage CAjan WT (lane 2). The expected product size is 1491 bp for the WT dpdA as observed in lane 2. The CAjan dpdAΔ PCR product is smaller due to the deletion, as we can observe in lane 1. The complete genomic sequence of the mutated phage was determined to confirm the absence of secondary mutations due to off targets.

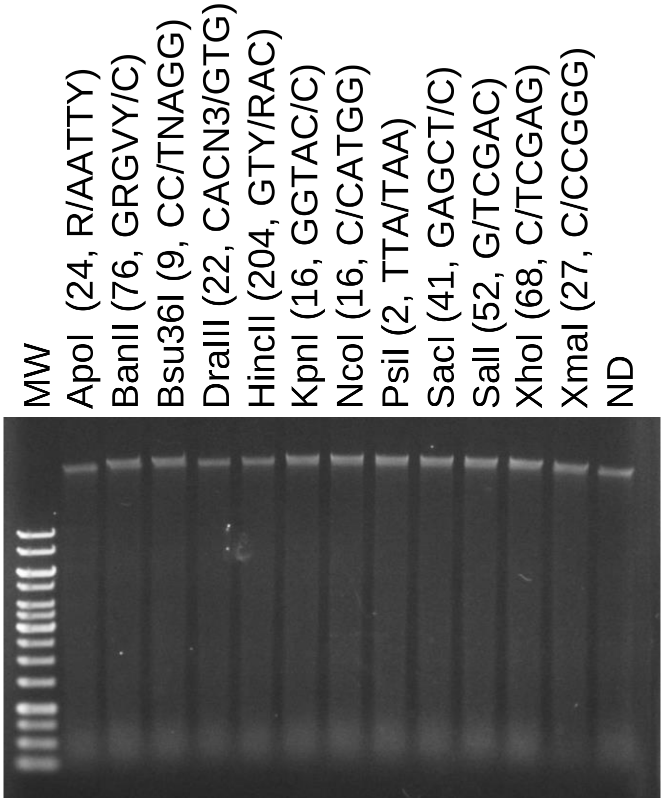

Supplementary Figure 4: Digestion of HVTV-1 DNA by 12 different restriction enzymes. Each line is annotated with the name of the enzyme and, in between parenthesis, the number of sites in HVTV-1 genome and the recognition site.

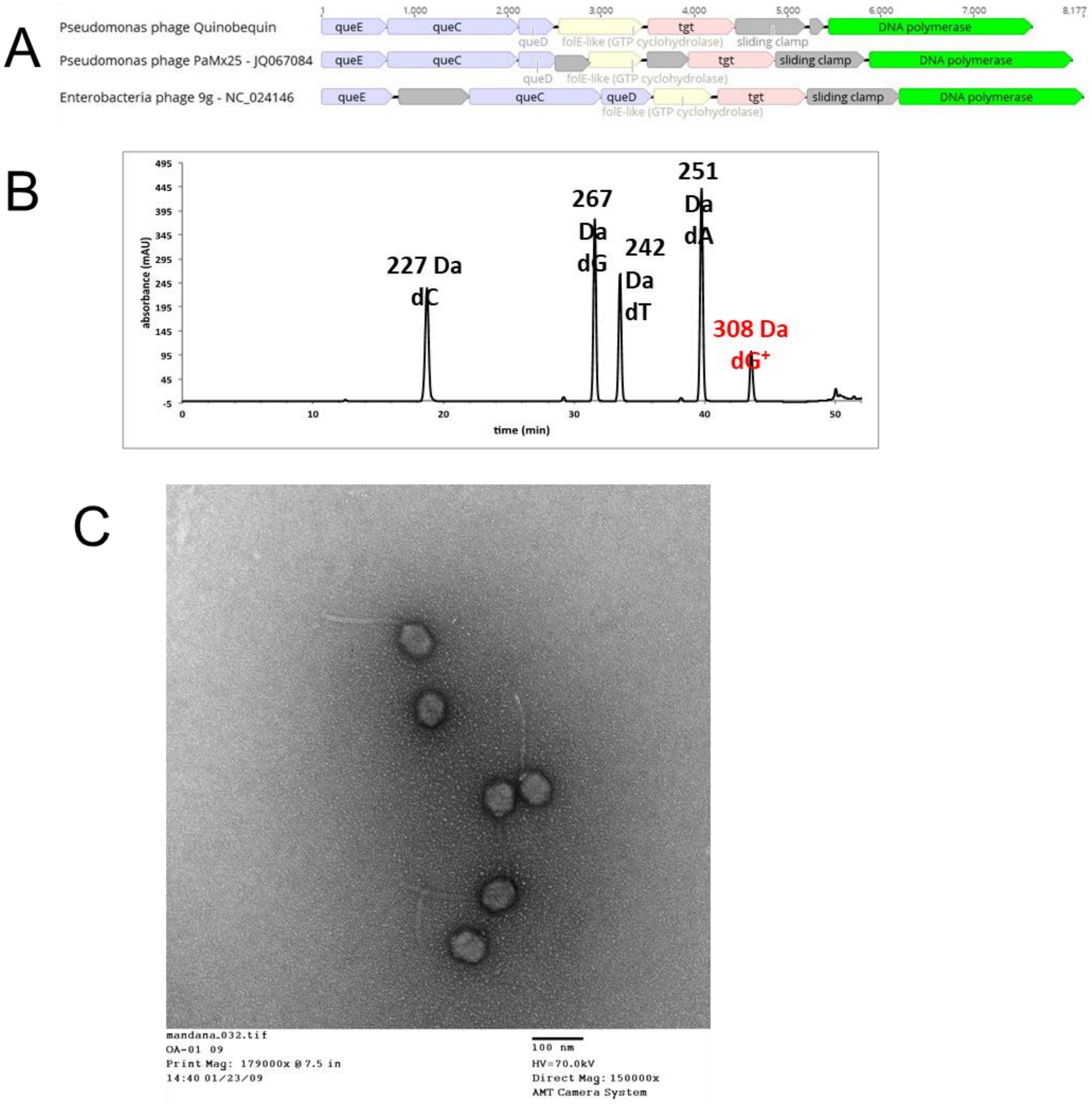

Supplementary Figure 5: *Pseudomonas* phages Quinobequin P09 analysis. A. Schematic showing the 2'-deoxyarchaeosine biosynthetic gene cluster of *Pseudomonas* phages Quinobequin P09 and PaMx25, as well as the *Enterobacteria* phage 9g **B.** The nucleoside composition of Quinobequin P09 is revealed by HPLC/MS analysis of enzymatic hydrolysate of purified virion DNA. Masses and nucleotide identities are indicated above detected peaks in the chromatogram. **C.** Electron microscopy of *Pseudomonas* phages Quinobequin P09.

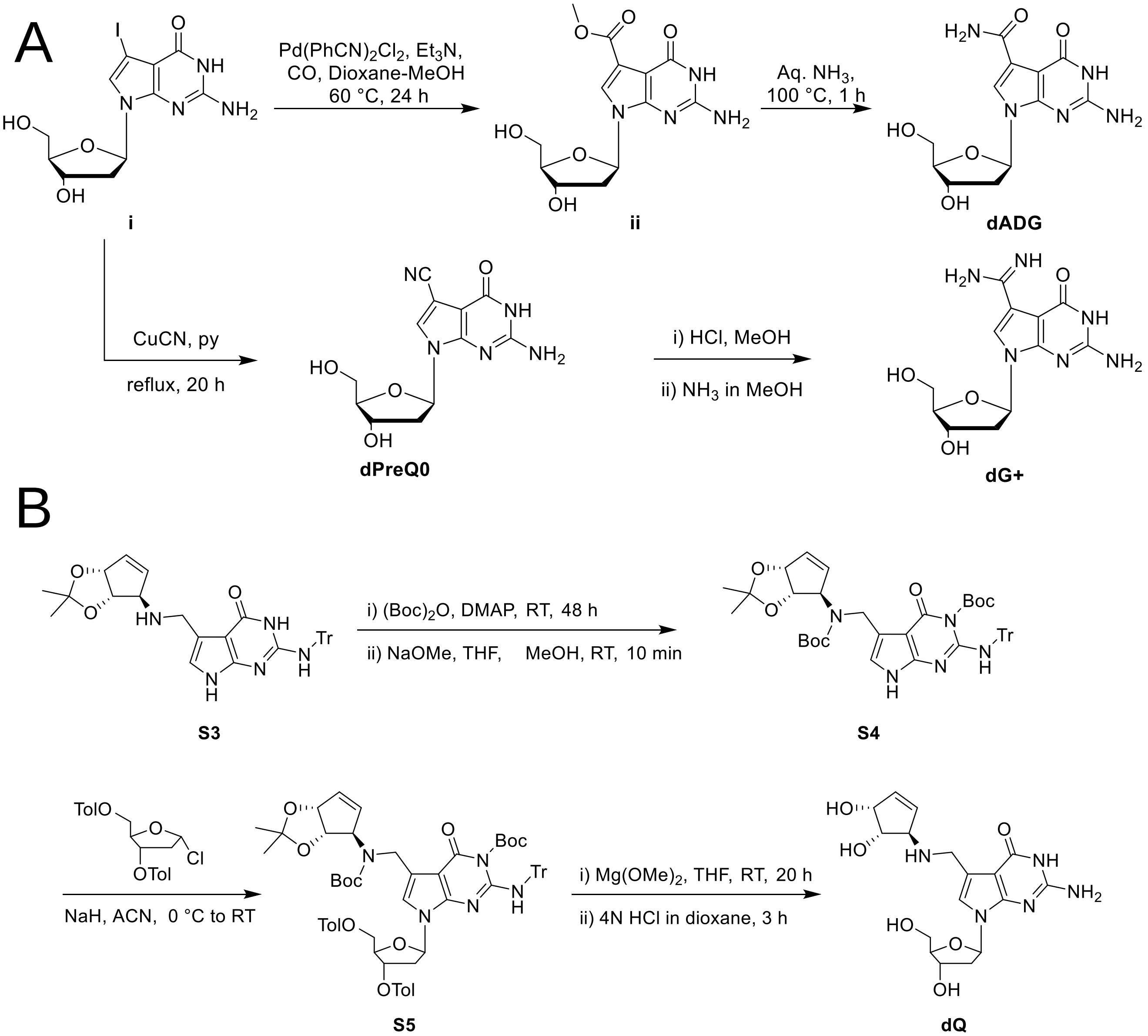

Supplementary Figure 6: Synthesis of the 2'deoxy-7-deazaguanine standards. A. Synthesis of dADG, $dPreQ_0$ and dG^{+} standards. The iodo compound (S1) was synthesized using procedures as previously reported[16] **B.** Synthesis of dQ; Compound S3 was synthesized using procedures as previously reported[20].

Supplementary Tables

accession #	protein name	species	EC number
WP_001139613	FolE	*Escherichia coli*	EC:3.5.4.16
WP_000987944	QueD	*Escherichia coli*	EC:4.1.2.50
WP_001199973	QueE	*Escherichia coli*	EC:4.3.99.3
WP_000817220	QueC	*Escherichia coli*	EC:6.3.4.20
WP_000100421	QueF	*Escherichia coli*	EC:1.7.1.13
WP_001266503	QueA	*Escherichia coli*	EC:2.4.99.17
WP_001294219	QueG	*Escherichia coli*	EC:1.17.99.6
WP_013679609	Gat-QueC	*Thermoproteus uzoniensis*	None - EC:6.3.4.20
BAA80469	QueF-L	*Aeropyrum pernix K1*	EC:2.6.1.-
WP_066380731	ArcS	*Halalkalicoccus paucihalophilus*	EC:2.6.1.97
WP_011068173	QueH	*Bifidobacterium longum* NCC2705	None
WP_005315061	DUF3820 (QueD-like)	*Aeromonas salmonicida*	None
YP_009032326	DpdA	*Enterobacteria* phage 9g	None
YP_008125322	DpdA2	*Vibrio* phage nt-1	None

Supplementary Table 1: Proteins use as anchors in the search for orthologues.

protein name	accession number in *Sulfolubus islandicus*
FolE	WP_012717327
QueD	WP_012712867
QueE	WP_014513981
Gat-QueC	WP_012712064
Arc-TGT	WP_012711831

Supplementary Table 2: G^+ biosynthesis proteins of *Sulfolobus islandicus* (tax id: 43080).

Plasmide name	Backbone	Insert	Reference
pBAD24	pBAD24	none	PMID:7608087
pCH111	pBAD24	*E. coli tgt*	This study
pGH39	pBAD24	9g *dpdA*	This study
pGH40	pBAD24	9g *folE*	This study
pGH41	pBAD24	9g *queD*	This study
pGH42	pBAD24	9g *gat-queC*	This study
pGH44	pBAD24	9g *queE*	This study
pBAD33	pBAD33	none	PMID:7608087
pGH65	pBAD33	9g *dpdA*	This study
pGH66	pBAD33	9g *gat-queC*	This study
pL2Cas9	pL2Cas9	none	PMID:28324650
pL2Cas9_dpdAΔ	pL2Cas9	spacer(TGCGGTCAAGCCAAGTCTTAAGCGTGTCCG)	This study
pNZ123	pNZ123	none	PMID:27143383
pNZ123_dpdAΔ	pNZ123	CAjan *dpdA* (del29212-29529)	This study

Supplementary Table 3: Plasmid list.

Supplementary references

1. Bertani, G. Studies on Lysogenesis I. The mode of phageliberation by lysogenic eschericia coli. *J. Bacteriol.* **62,** 293–300 (1951).
2. RL, H. Elective localization in the eye of bacteria from infected teeth. *Arch. Intern. Med.* **32,** 828–849 (1923).
3. Ouennane, S., Leprohon, P. & Moineau, S. Diverse virulent pneumophages infect Streptococcus mitis. *PLoS One* **10,** 1–14 (2015).
4. Baba, T. *et al.* Construction of Escherichia coli K-12 in-frame, single-gene knockout mutants: the Keio collection. *Mol. Syst. Biol.* **2,** 2006.0008 (2006).
5. Moore, S. D. Assembling New Escherichia coli Strains by Transduction Using Phage P1. in *Strain Engineering: Methods and Protocols* (ed. Williams, J. A.) 155–169 (Humana Press, 2011). doi:10.1007/978-1-61779-197-0_10
6. Datsenko, K. a & Wanner, B. L. One-step inactivation of chromosomal genes in Escherichia coli K-12 using PCR products. *Proc. Natl. Acad. Sci. U. S. A.* **97,** 6640–6645 (2000).
7. Lee, D. G. *et al.* Genomic analysis reveals that Pseudomonas aeruginosa virulence is combinatorial. *Genome Biol.* **7,** (2006).
8. Van Twest, R. & Kropinski, A. M. Bacteriophage Enrichment from Water and Soil. in *Bacteriophages: Methods and Protocols, Volume 1: Isolation, Characterization, and Interactions* (eds. Clokie, M. R. J. & Kropinski, A. M.) 15–21 (Humana Press, 2009). doi:10.1007/978-1-60327-164-6_2
9. Lee, Y.-J. *et al.* Identification and biosynthesis of thymidine hypermodifications in the genomic DNA of widespread bacterial viruses. *Proc. Natl. Acad. Sci.* 201714812 (2018). doi:10.1073/pnas.1714812115
10. Roberts, R. J., Carneiro, M. O. & Schatz, M. C. The advantages of SMRT sequencing. *Genome Biol.* **14,** 6–9 (2013).
11. Aziz, R. K. *et al.* The RAST Server: Rapid annotations using subsystems technology. *BMC Genomics* **9,** 1–15 (2008).
12. Thiaville, J. J. *et al.* Novel genomic island modifies DNA with 7-deazaguanine derivatives. *Proc. Natl. Acad. Sci. U. S. A.* **113,** E1452-9 (2016).
13. Sarkis, G. J. & Hatfull, G. F. Mycobacteriophages. in *Mycobacteria Protocols* (eds. Parish, T. & Stoker, N. G.) 145–173 (Humana Press, 1998). doi:10.1385/0-89603-471-2:145
14. Eskelin, K. *et al.* Halophilic viruses with varying biochemical and biophysical properties are amenable to purification with asymmetrical flow field-flow fractionation. *Extremophiles* **21,** 1119–1132 (2017).
15. Gibson, D. G. *et al.* Enzymatic assembly of DNA molecules up to several hundred kilobases. *Nat. Methods* **6,** 343–345 (2009).
16. Ingale, S. A. & Seela, F. Nucleoside and oligonucleotide pyrene conjugates with 1,2,3-triazolyl or ethynyl linkers: Synthesis, duplex stability, and fluorescence changes generated by the DNA-dye connector. *Tetrahedron* **70,** 380–391 (2014).

17. Ramzaeva, N., Becher, G. & Seela, F. *Facile synthesis of 2'-deoxynucleoside analogs of preQ. Synthesis* (Thieme Chemistry, 1998).
18. Brückl, T., Klepper, F., Gutsmiedl, K. & Carell, T. A short and efficient synthesis of the tRNA nucleosides PreQ0 and archaeosine. *Org. Biomol. Chem.* **5,** 3821–3825 (2007).
19. Wang, R. W. & Gold, B. A facile synthetic approach to 7-deazaguanine nucleosides via a boc protection strategy. *Org. Lett.* **11,** 2465–2468 (2009).
20. Brooks, A. F., Garcia, G. A. & Showalter, H. D. H. A short, concise synthesis of queuine. *Tetrahedron Lett.* **51,** 4163–4165 (2010).
