# Supplementary material for: 7-Deazaguanine modifications protect phage DNA from host restriction systems
Source: Nat Commun. 2019 Nov 29;10:5442. doi: 10.1038/s41467-019-13384-y (PMC6884629; doi:10.1038/s41467-019-13384-y)
Supplement: Supplementary file 4 — Description of Additional Supplementary Files [file 41467_2019_13384_MOESM4_ESM.docx]

**Description of Additional Supplementary Files**

File name: Supplementary Data 1
Description: Proteins identified in viruses.

File name: Supplementary Data 2
Description: Viruses classifications and their hosts.

File name: Supplementary Data 3
Description: Q and G^+^ biosynthesis gene content of the hosts.

File name: Supplementary Data 4
Description: Strain list.

File name: Supplementary Data 5
Description: Phage list.

File name: Supplementary Data 6
Description: Oligonucleotide list.
