# Supplementary material for: 7-Deazaguanine modifications protect phage DNA from host restriction systems
Source: Nat Commun. 2019 Nov 29;10:5442. doi: 10.1038/s41467-019-13384-y (PMC6884629; doi:10.1038/s41467-019-13384-y)
Supplement: Supplementary file 11 — Source Data [file 41467_2019_13384_MOESM11_ESM.zip › Source data-corrected.pptx]

## Slide 1
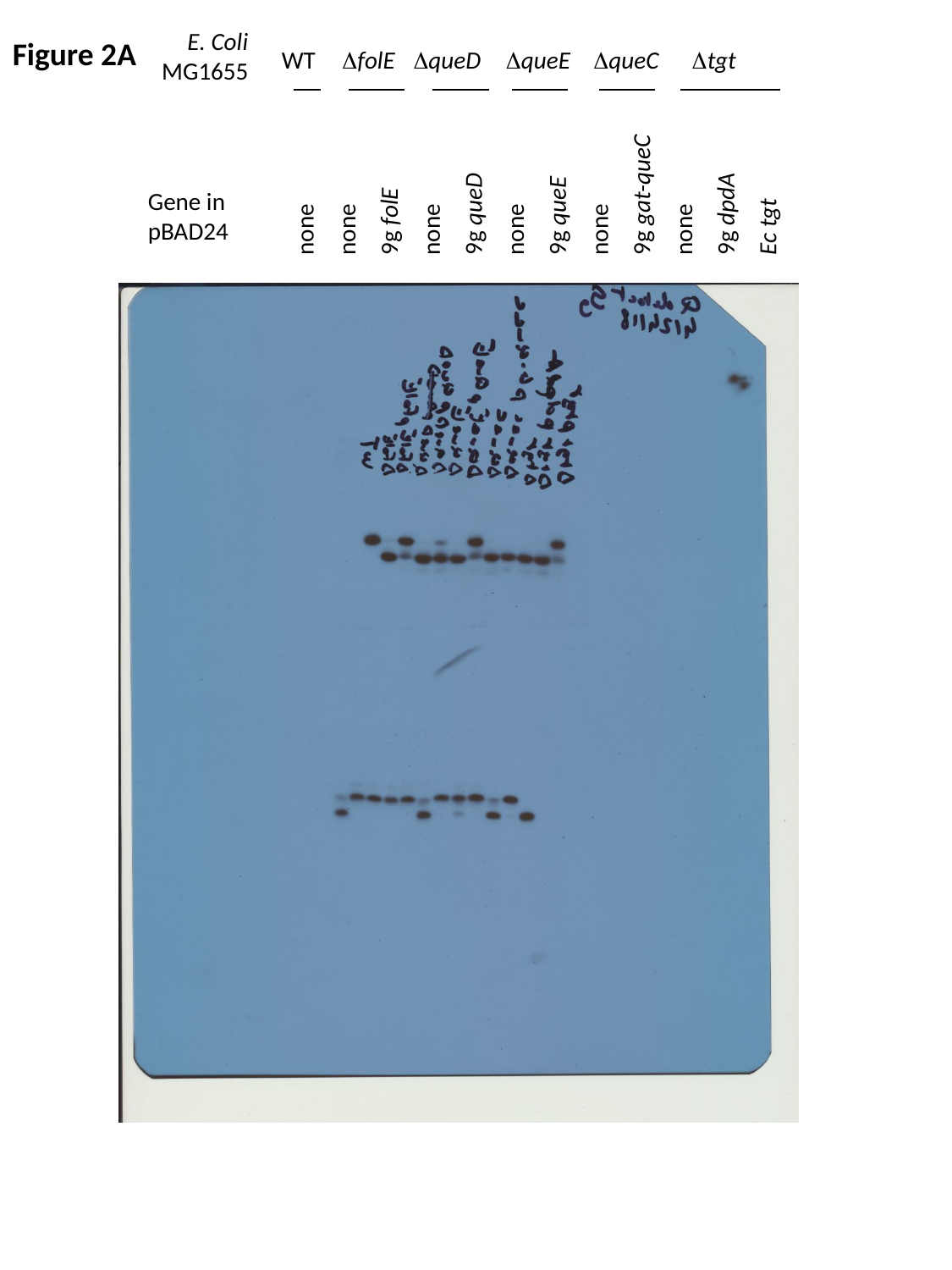

E. Coli MG1655
Figure 2A
WT
DfolE
DqueD
DqueE
DqueC
Dtgt
9g gat-queC
9g queD
9g queE
9g dpdA
Gene in pBAD24
9g folE
none
none
none
none
none
none
Ec tgt

## Slide 2
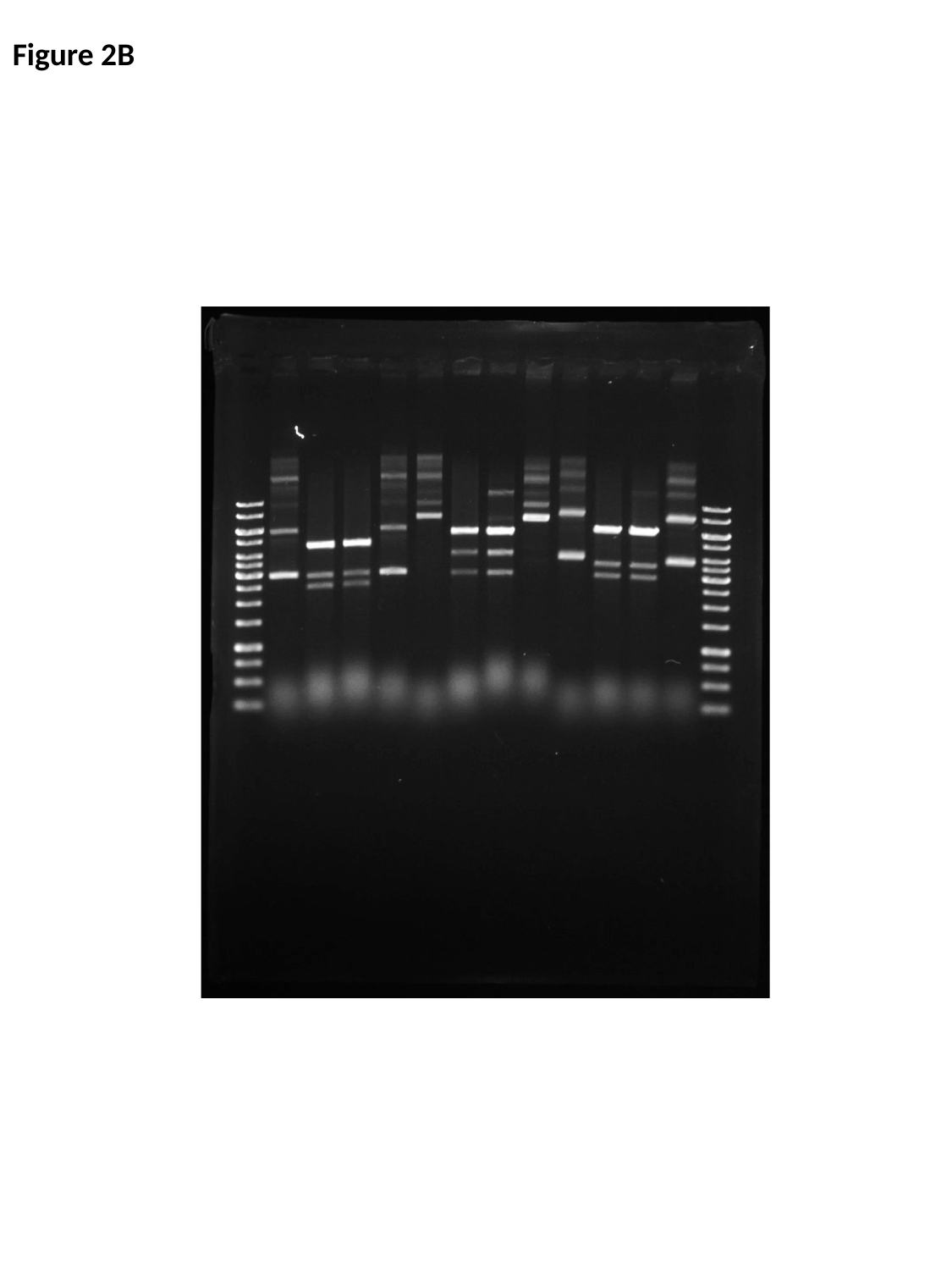

Figure 2B

## Slide 3
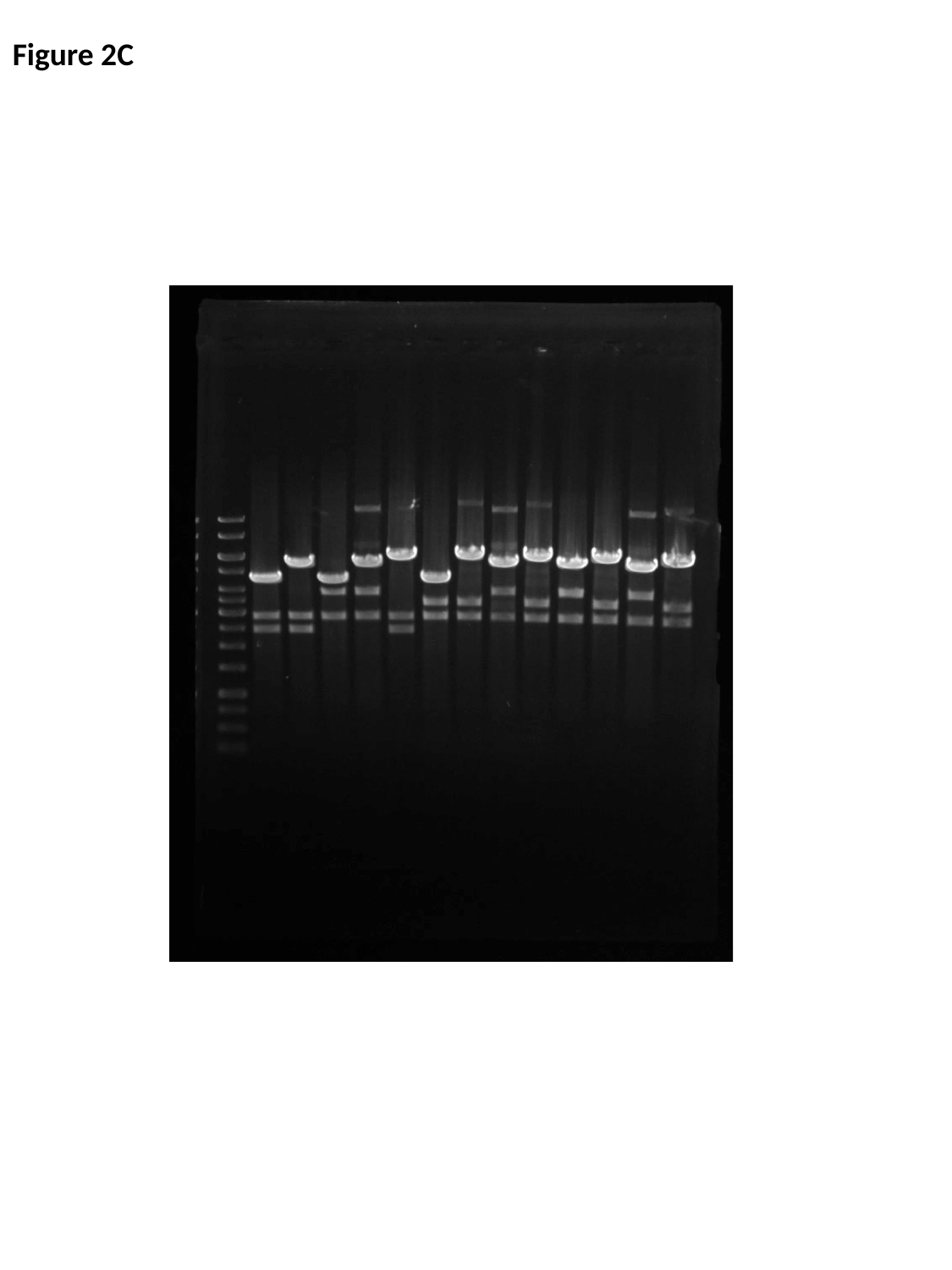

Figure 2C

## Slide 4
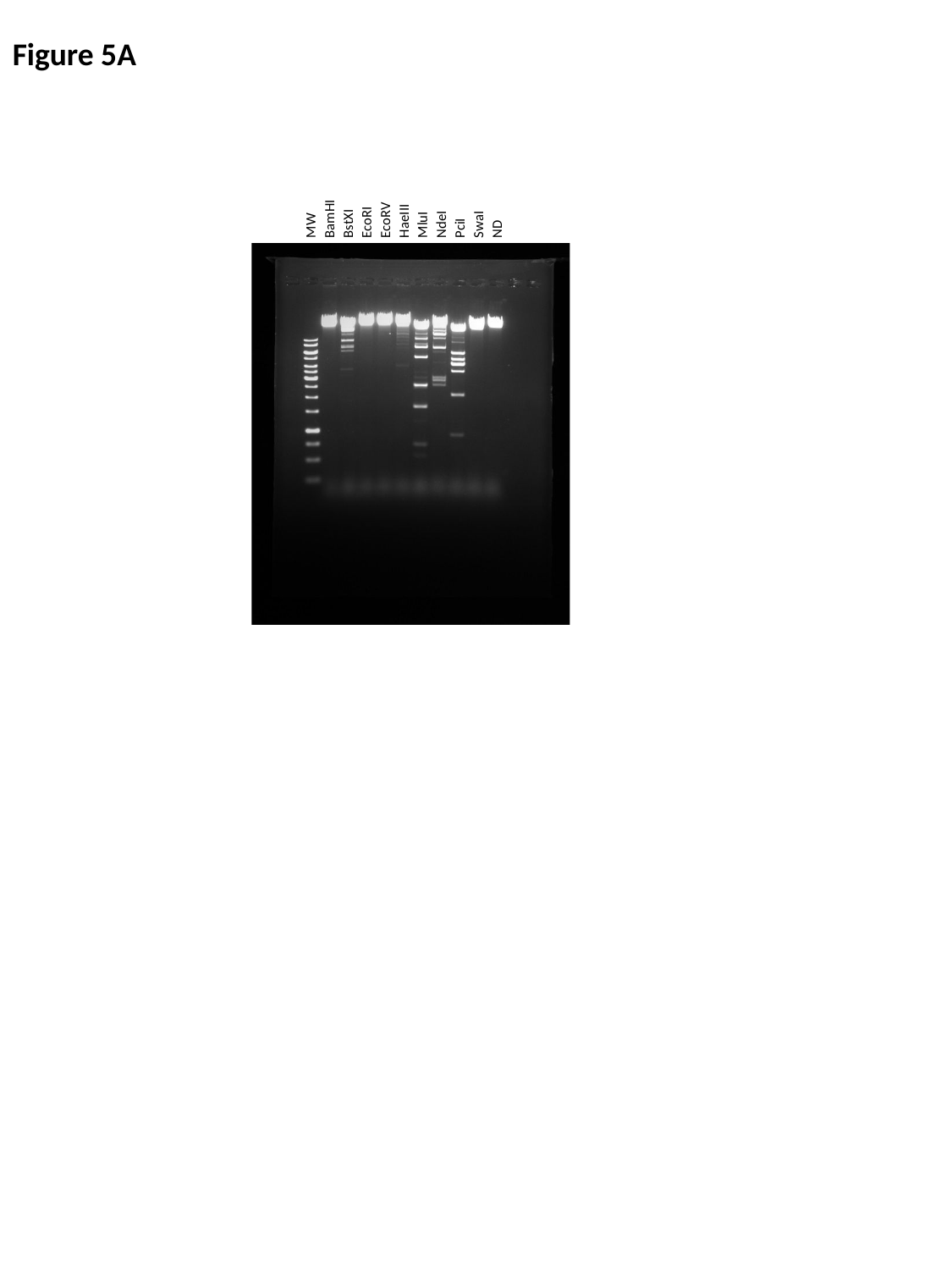

Figure 5A
MW
BamHI
BstXI
EcoRI
EcoRV
HaeIII
MluI
NdeI
PciI
SwaI
ND

## Slide 5
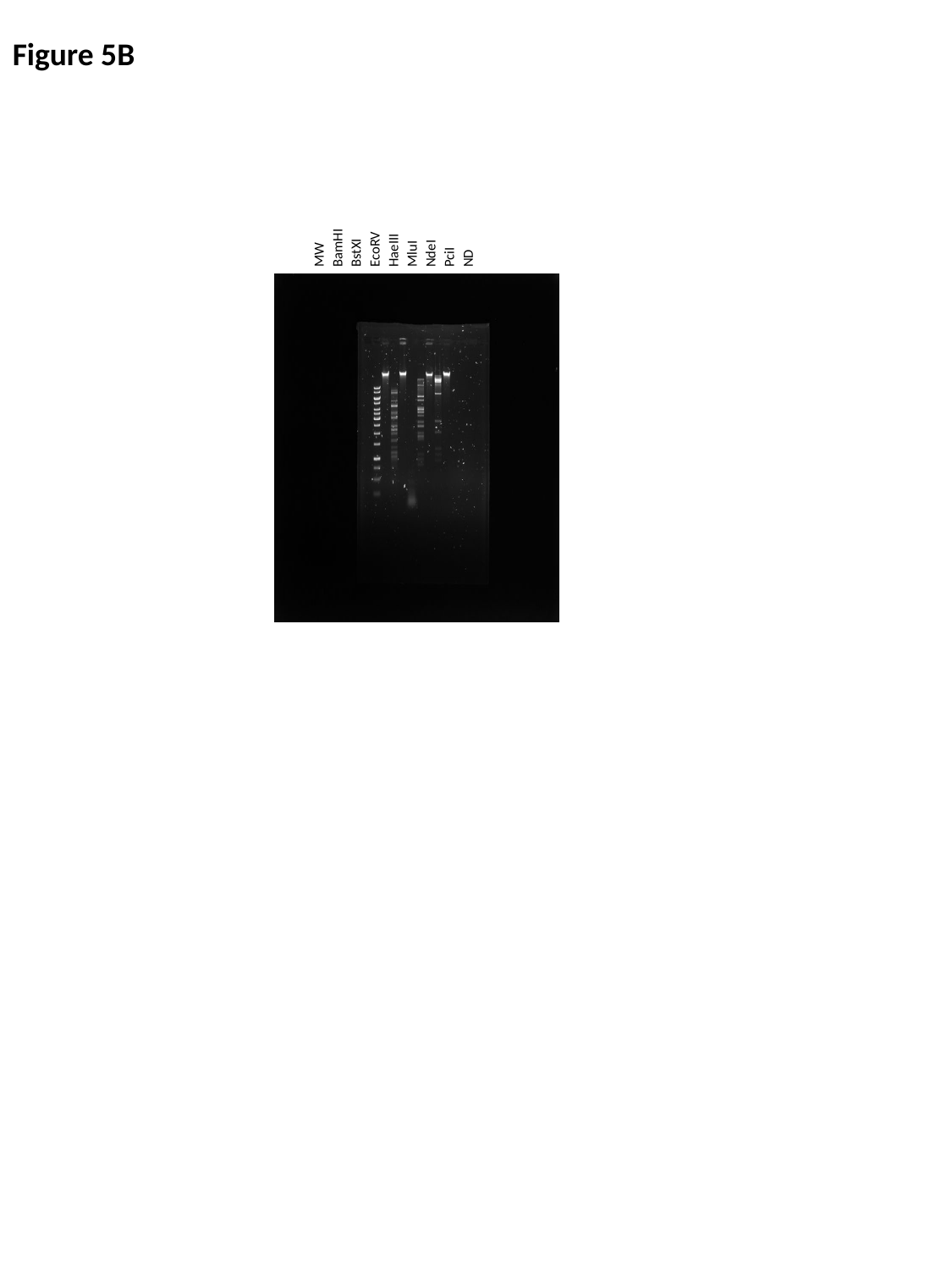

Figure 5B
MW
BamHI
BstXI
EcoRV
HaeIII
MluI
NdeI
PciI
ND

## Slide 6
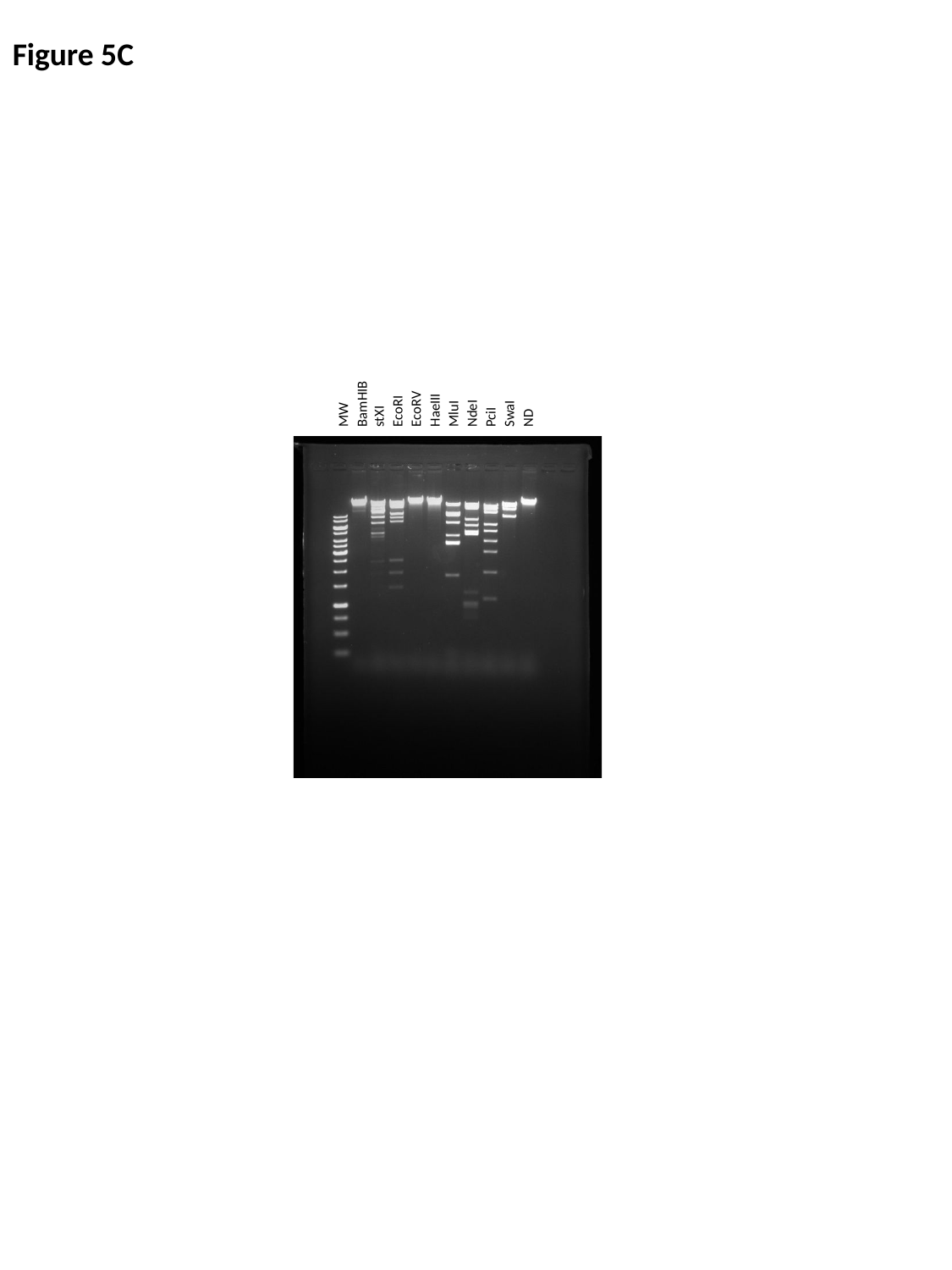

Figure 5C
MW
BamHIBstXI
EcoRI
EcoRV
HaeIII
MluI
NdeI
PciI
SwaI
ND

## Slide 7
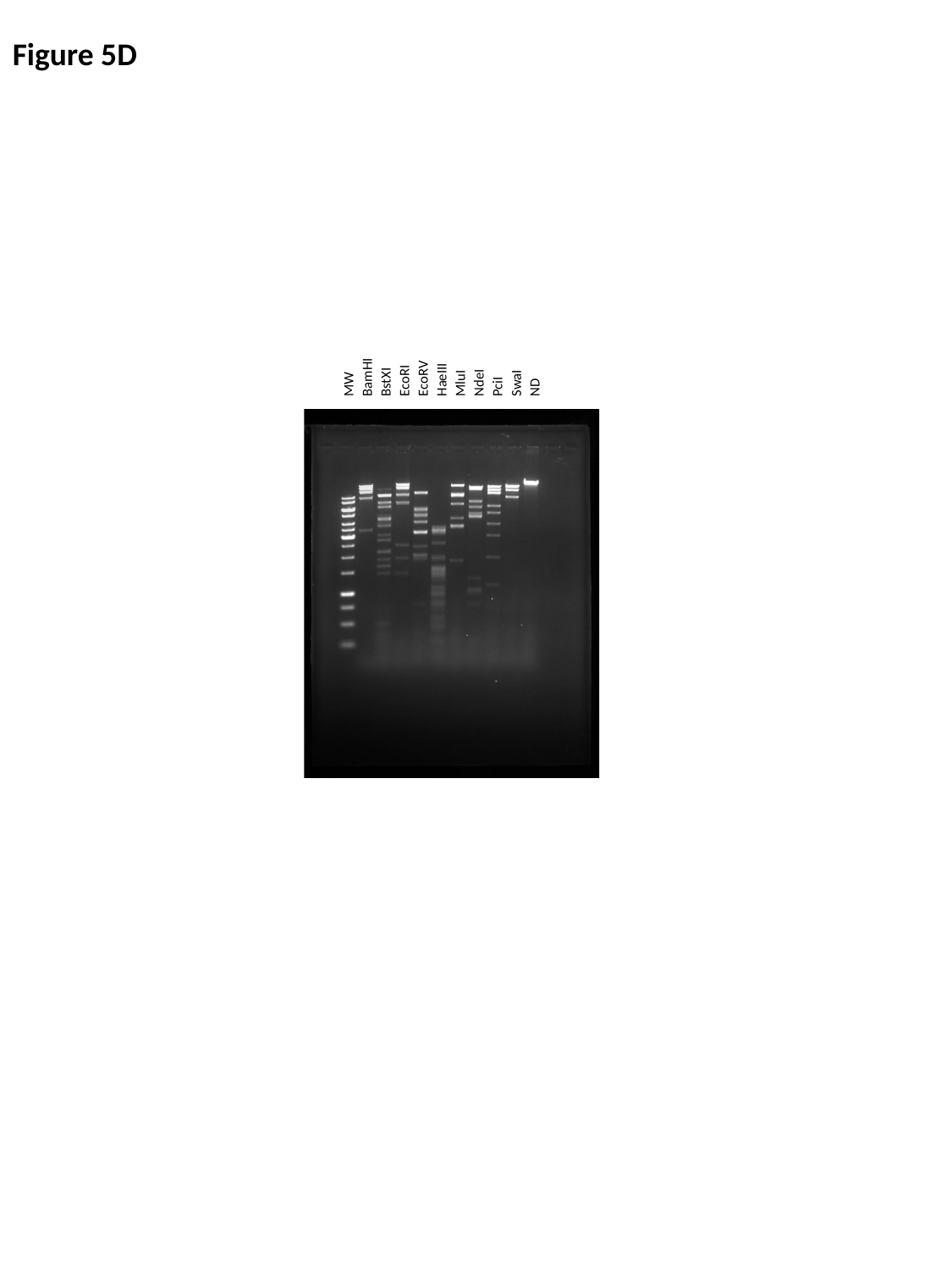

Figure 5D
MW
BamHI
BstXI
EcoRI
EcoRV
HaeIII
MluI
NdeI
PciI
SwaI
ND

## Slide 8
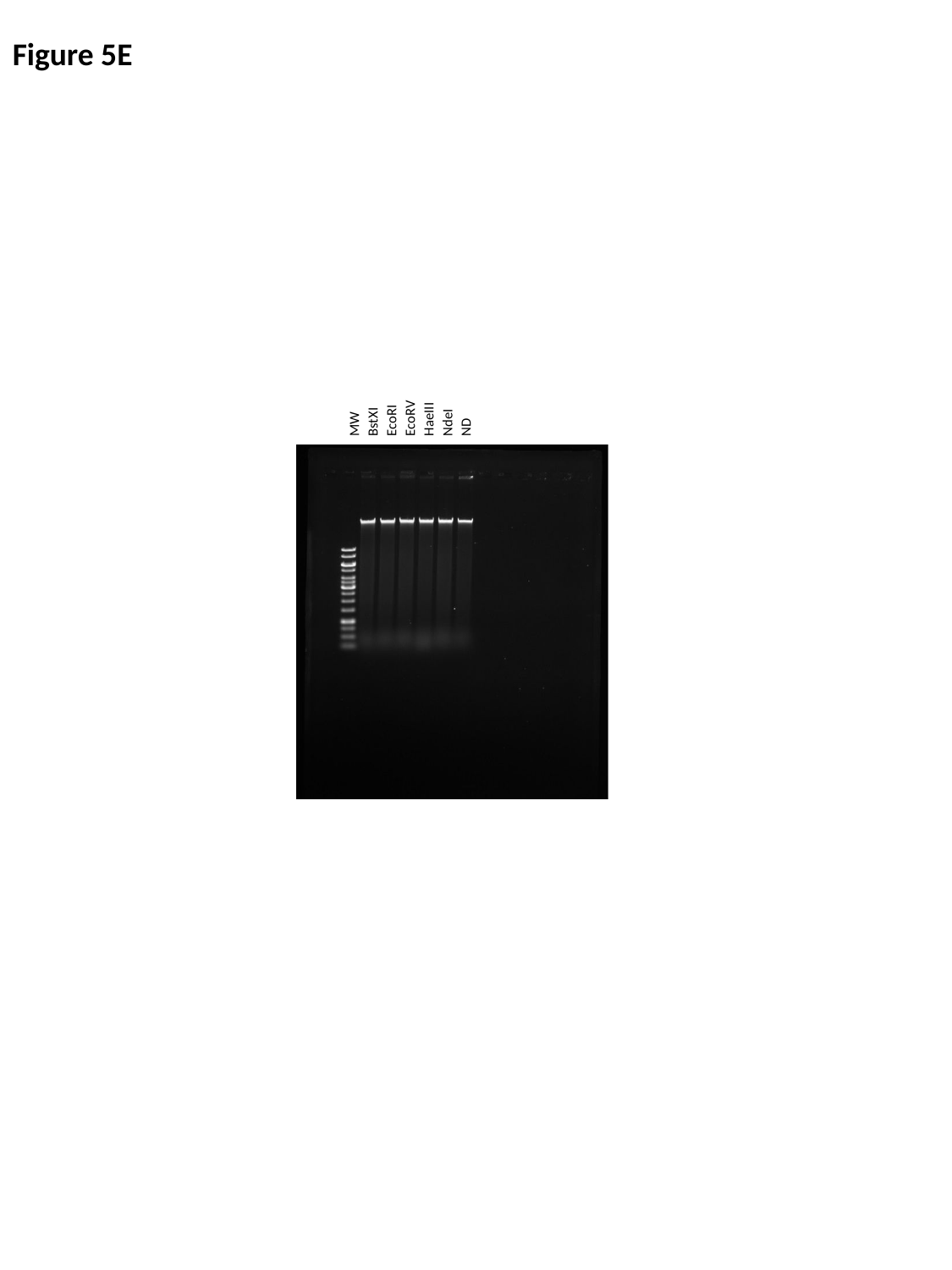

Figure 5E
MW
BstXI
EcoRI
EcoRV
HaeIII
NdeI
ND

## Slide 9
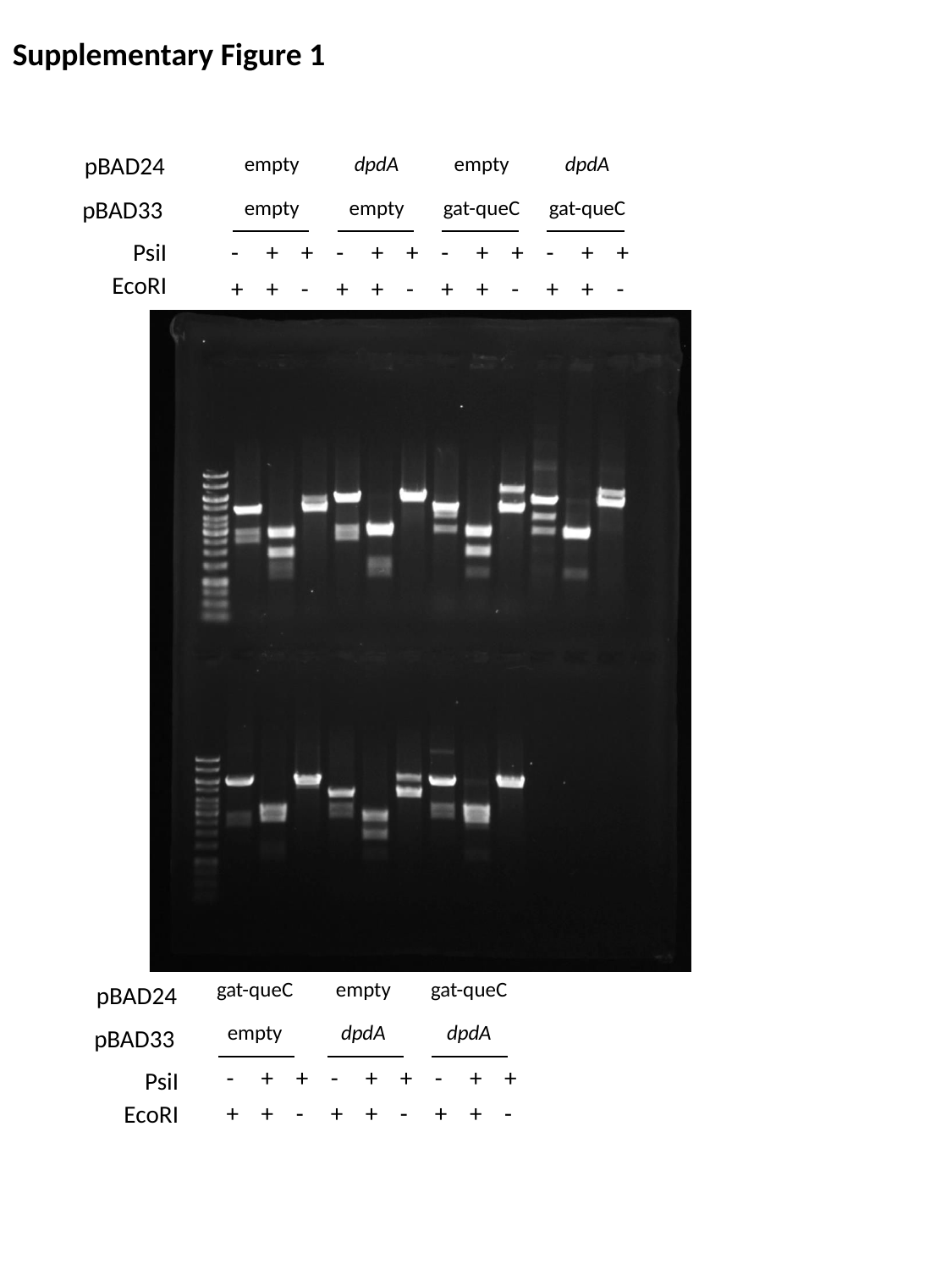

Supplementary Figure 1
pBAD24
empty
dpdA
empty
dpdA
pBAD33
empty
empty
gat-queC
gat-queC
PsiI
-
+
+
-
+
+
-
+
+
-
+
+
EcoRI
+
+
-
+
+
-
+
+
-
+
+
-
gat-queC
empty
gat-queC
pBAD24
empty
dpdA
dpdA
pBAD33
-
+
+
-
+
+
-
+
+
PsiI
+
+
-
+
+
-
+
+
-
EcoRI

## Slide 10
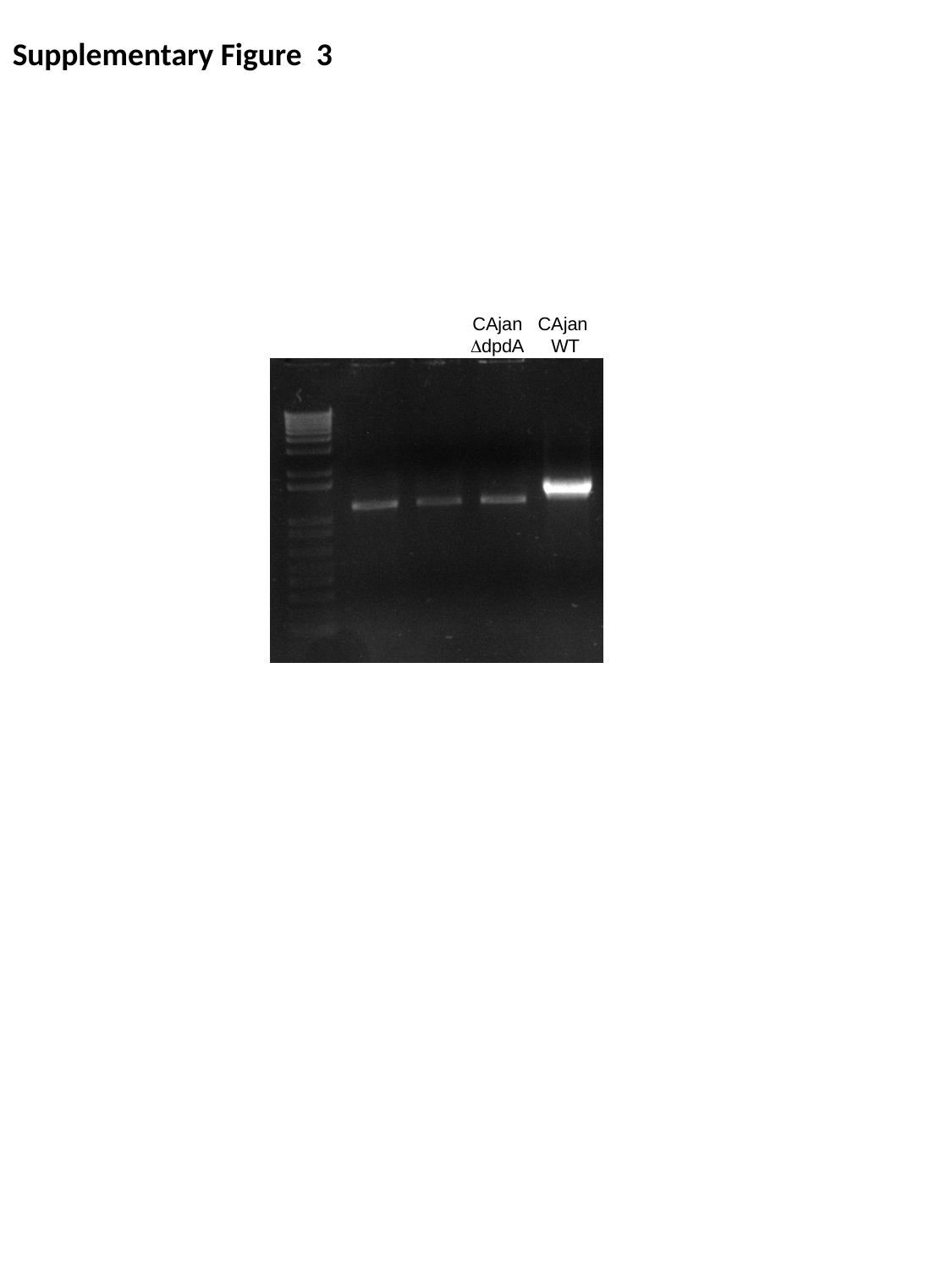

Supplementary Figure 3
CAjan
DdpdA
CAjan
WT

## Slide 11
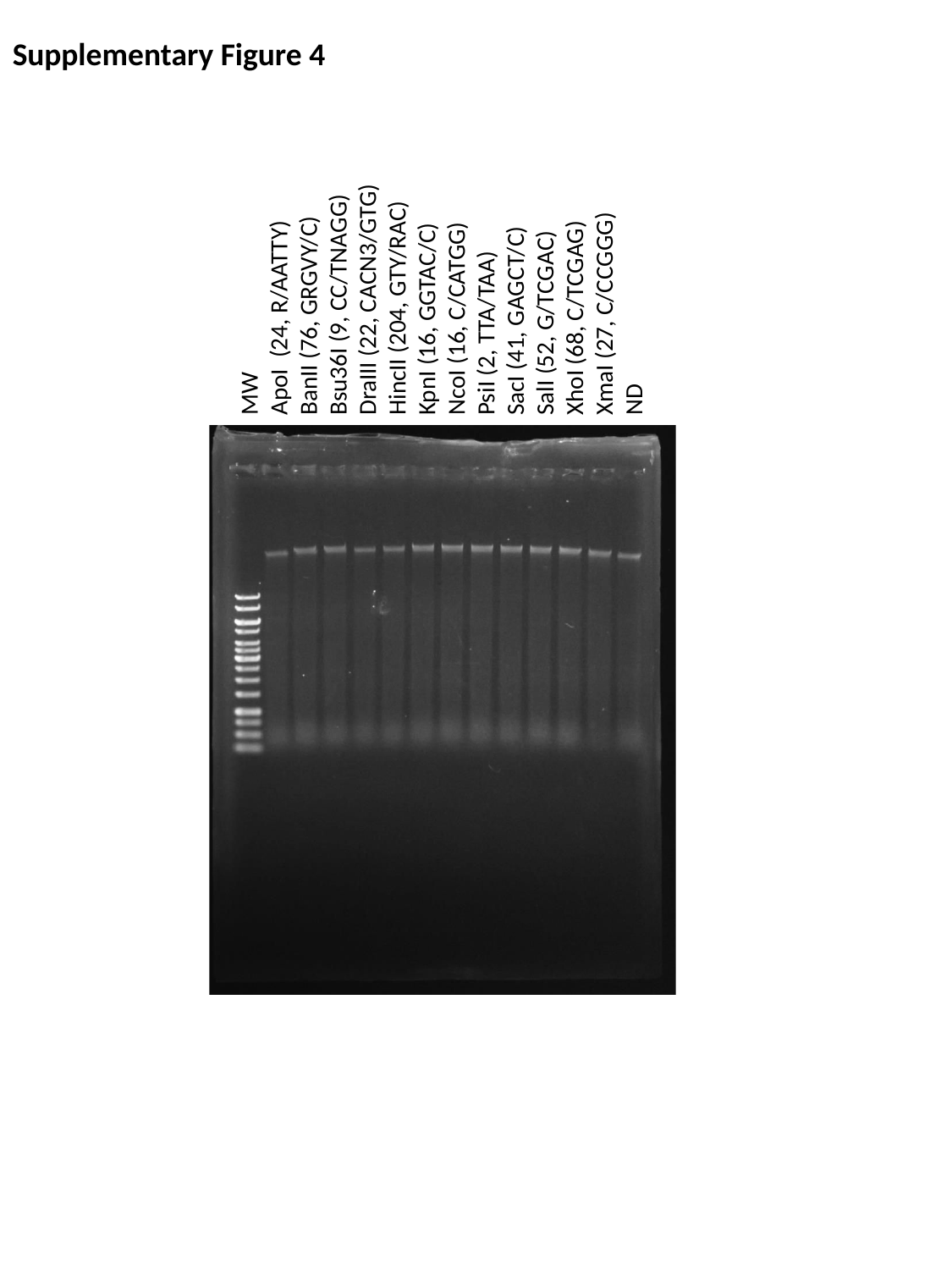

Supplementary Figure 4
MW
ApoI (24, R/AATTY)
BanII (76, GRGVY/C)
Bsu36I (9, CC/TNAGG)
DraIII (22, CACN3/GTG)
HincII (204, GTY/RAC)
KpnI (16, GGTAC/C)
NcoI (16, C/CATGG)
PsiI (2, TTA/TAA)
SacI (41, GAGCT/C)
SalI (52, G/TCGAC)
XhoI (68, C/TCGAG)
XmaI (27, C/CCGGG)
ND
