# Supplementary material for: 7-Deazaguanine modifications protect phage DNA from host restriction systems
Source: Nat Commun. 2019 Nov 29;10:5442. doi: 10.1038/s41467-019-13384-y (PMC6884629; doi:10.1038/s41467-019-13384-y)
Supplement: Supplementary file 2 — Peer Review [file 41467_2019_13384_MOESM2_ESM.pdf]

Reviewers' comments:

Reviewer #1

This paper reports on a pathway by which 7-deazaguanine bases are inserted into both phage and host DNA. The modifications would protect the phage DNA from cleavage by host-encoded restriction endonucleases. This is very interesting work with broad implications for competition between hosts and phages.

The work is well done from a technical perspective. The manuscript is somewhat hard to follow if one is unfamiliar with the naming schemes. The significance of the paper is the newly identified DNA modifications. A figure outlining that pathway does not occur until Fig. 6 in the discussion.

The unusual step in the pathway is the "insertase" nature of the enzymes that place the modified purine-mimic base into the intact DNA strand. More discussion of this activity is needed as it is very unusual.

While guanine substitution would block many restriction enzymes, the described modification would also block the binding of other sequence-specific DNA-binding proteins such as transcription factors. The implications for these interactions, as well as potential sequence-specificity should be addressed.

Larry Sowers

Reviewer #2

This paper reports the cloning and expression of dG+ biosynthesis pathway from phage 9g in laboratory E. coli strain and uncovers the individual steps in the dG+ modification reactions by deletion and complementation analysis. By combination of comparative genomics and individual gene analysis, and mass spectrometry analysis the authors discovered additional phages with dG+ modification. They also reported hypermodified bases dPreQ0 in Escherichia phage CAjan and Mycobacterium phage Rosebush, and modified dPreQ1 in Halovirus HVTV-1. In addition, the modified base dADG was also detected in a number of phage genomes, although the modification level is not as high as dG+, dPreQ0 or dPreQ1.
I have a few comments below:
1. In results and Figure 2. The expression level and dG+ biosynthesis is inferred from plasmid resistance to EcoRI digestion due to incorporated dG+ in GAATTC sites and further verified by LC-MS analysis. What is the estimated ratio of dG+/Gs in the plasmid and genomic DNA under induced condition? Did it reach as high as 25% as in the phage genome? It might be a good control to choose another restriction enzyme that cuts A/T sequence and digest the EcoRI-resistant fragment.
2. In results and Figure 5. HVTV-1 DNA is highly resistant to Type II restrictions. Is there any restriction enzyme that can cleave this DNA? For example, MluCI (AATT). Just to make sure the prepared gDNA is clean and truly resistant.
3. To gain advantage, the phage genomes reported in this work "invented" modified G (7-deazaG derivatives) to protect phage genomes against restrictions. However, bacterial hosts evolve modification-dependent restriction systems to attack modified phage genomes. Examples are SRA-HNH and PvuRts1I family of modification-dependent restriction systems that can restrict 5hmC-modified phage DNA, and GmrSD family of enzymes restricting glucosylated-5hmC phage genomes. This paper highlights many hypermodified phage genomes in the phage-host biological arms race. But the next generation of arsenal- potential modification-dependent restriction systems to restrict/attack these modified DNA is missing in the discussion.
Other minor points:
1. The order of Figure 2B and 2C presentation is reversed. (2C presented first).
2. Figure legend, page 25. Figure 7 should be Figure 6.

3. In a few places, restriction digestions carried out in 20 ul not in 20 ml.
4. Phage 34. Figure 3. dPreQ0 pathway. LC-MS confirmed that it is dPreQ0.
5. What other predicted DNA modification genes are present in the Halovirus HVTV-1 genome?
6. Figure 4. Are the protein networks supposed to be readable by the readers? Apparently some of the nodes are too small to be eligible.
7. Page 22. “Washed”.
8. Supplement material.

Page 2. line 61. Species name should be in italic.
Page 3. line 126, MgCl2, line 128, 55oC.
Page 4. Lines 140 and 141, fix MgCl2, MgSO4, CaCl2. Line 158, fix dH2O. line 165, fix MgSO4.
Page 5. Line 182, Fix dH2O. line 186, fix 2ul. Line 186 and 187, check oligo concentration 50mM.
Page 9, DNA pol ge

Reviewer #3

This is an interesting paper which extends recent discoveries of non-canonical bases in phage DNA, identifying three new modifications. Functionality is also demonstrated in terms of providing resistance to host restriction enzymes.
The language is quite dense and can be difficult to follow the thread of the argument. Numbered lanes are referred to in the text; e.g. lines 113 and 116), but are not numbered in Figure 2. Figures 2B and 2C seem to be reversed in the text. Similarly, bands are referred to (uncut plasmid) but not indicated in the figure so there is a bit of guesswork involved.
Paragraph (lines 142 – 147) is not clear, please re-word.
Line 152; some words missing?
Figure 4 is of limited value to the reader, and could be moved to Supplementary.
I am not convinced about the link between modification and phage infecting pathogens - is this due to an overabundance of pathogenic bacteriophages in databases, or is it ‘real’?
Lines 273-275 are speculative, not experimental results, and should be moved to the Discussion.

Reviewer 1

This paper reports on a pathway by which 7-deazaguanine bases are inserted into both phage and host DNA. The modifications would protect the phage DNA from cleavage by host-encoded restriction endonucleases. This is very interesting work with broad implications for competition between hosts and phages.

The work is well done from a technical perspective. The manuscript is somewhat hard to follow if one is unfamiliar with the naming schemes. The significance of the paper is the newly identified DNA modifications.
A figure outlining that pathway does not occur until Fig. 6 in the discussion.
The DNA modification pathway is presented only in the last figure for two reasons. First, the tRNA modification pathway presented in Figure 1 is very similar to the DNA modification pathway. Only one step is different, the insertion of the modified base in DNA. Second, this paper describes the predictions of the genes involved in the modifications identified in the different phages; the last figure summarizes these predictions based on our knowledge of the tRNA modification pathways.

The unusual step in the pathway is the "insertase" nature of the enzymes that place the modified purine-mimic base into the intact DNA strand. More discussion of this activity is needed as it is very unusual.
The statement above was already discussed as followed: “As with its bacterial homolog[27], the phage DpdA introduces $PreQ_0$ in DNA (**Figure 2C, Table1**), most probably through a base exchange mechanism similar to its TGT homolog[31]. DpdA2 proteins appear to share this function, as *Vibrio* phage nt-1 genome contains $dPreQ_0$”.
We chose not to use the name “insertase” as it is mostly used to describe proteins that insert proteins in the membrane. We used the name “2’-deoxyribosyltransferase” later in the text. As the exact biochemistry and position of the base transfer are not known, it is difficult to elaborate further. Further studies are required to pursue the characterization of this activity.

While guanine substitution would block many restriction enzymes, the described modification would also block the binding of other sequence-specific DNA-binding proteins such as transcription factors. The implications for these interactions, as well as potential sequence-specificity should be addressed.
We addressed these concerns in the discussion as followed: “These modifications might also block other DNA binding proteins that require the nitrogen moiety at position 7 of the guanine to recognize its substrate, the most critical being sigma and transcription factors. However, it is to be noted that phages only use the housekeeping sigma factor[49], that has an AT-rich recognition sequence[50], and encodes their own transcription factors.”

Larry Sowers

Reviewer 2

This paper reports the cloning and expression of dG+ biosynthesis pathway from phage 9g in laboratory E. coli strain and uncovers the individual steps in the dG+ modification reactions by deletion and complementation analysis. By combination of comparative genomics and individual gene analysis, and mass spectrometry analysis the authors discovered additional phages with dG+ modification. They also reported hypermodified bases dPreQ0 in Escherichia phage CAjan and Mycobacterium phage Rosebush, and modified dPreQ1 in Halovirus HVTV-1. In addition, the modified base dADG was also detected in a number of phage genomes, although the modification level is not as high as dG+, dPreQ0 or dPreQ1.
I have a few comments below:
1. In results and Figure 2. The expression level and dG+ biosynthesis is inferred from plasmid resistance to EcoRI digestion due to incorporated dG+ in GAATTC sites and further verified by LC-MS analysis. What is the estimated ratio of dG+/Gs in the plasmid and genomic DNA under induced condition? Did it reach as high as 25% as in the phage genome? It might be a good control to choose another restriction enzyme that cuts A/T sequence and digest the EcoRI-resistant fragment.
We inserted the quantification of the amount of modifications both in a new table (Table 1) and in the text, as followed :"Analysis of dG^+, dADG, $dPreQ_0$ and $dPreQ_1$ profiles by liquid chromatography-coupled triple quadrupole mass spectrometry (LC-MS/MS, quantification results in **Table 1**) revealed that plasmid DNA extracted from strains expressing only *dpdA* contained $dPreQ_0$, with 790 ± 8 modifications per 10^6 nucleotides, 0.316 ± 0.0032 % of the Gs, when expressed in pBAD24 and 84 ± 26 modifications per 10^6 nucleotides, 0.0336 ± 0.0104 % of the Gs, when expressed in pBAD33. A just above detection level of dG^+ was also detected in this strain (6.5 ± 0.5 modifications per 10^6 nucleotides, 0.0026 ± 0.0002 % of the Gs). Plasmid DNA extracted from strains expressing *dpdA* and *gat-queC* contained dG^+, with 45000 ± 25000 modifications per 10^6 nucleotides, 18 ± 10 % of the Gs, when DpdA was expressed in pBAD24 and Gat-QueC in pBAD33, 22750 ± 17250 modifications per 10^6 nucleotides, 9.1 ± 7 % of the Gs, when reversed. $dPreQ_0$ was also detected when *gat-queC* was expressed at lower levels than *dpdA*, (77 ± 7 modifications per 10^6 nucleotides, 0.0308 ± 0.0028 % of the Gs). No modifications were detected in strains harboring empty plasmids or when only Gat-QueC was expressed (**Table 1**)" and then later: "Furthermore, only dG^+ modification was observed in DNA of the *ΔqueC* strains by LC-MS/MS (**Table 1**), with similar amounts than in the WT (13750 modifications per 10^6 nucleotides, 5.5 % of the Gs, and 23000±17000 modifications per 10^6 nucleotides, 9.2 ± 7% of the Gs)".
We also added a supplemental figure (Supplemental Figure 1) showing the double digestions of the plasmids with EcoRI and PsiI (TTA^TAA). The double digestion of the modified plasmids showed a supplementary band that correspond to the PsiI linearized plasmids which are not digested by EcoRI, thus protected from restriction by the modification.
The text has been modified as followed: "As a supplemental control, we digested the same combination of plasmids with PsiI (TTA^TAA) and EcoRI (**Supplemental Figure 1**). The single digestion by PsiI linearized all these plasmids, and the plasmids encoding for both *dpdA* and *gat-queC* of phage 9g was again partially resistant to EcoRI digestion (red arrows in **Supplemental Figure 1**)".

2. In results and Figure 5. HVTV-1 DNA is highly resistant to Type II restrictions. Is there any restriction enzyme that can cleave this DNA? For example, MluCI (AATT). Just to make sure the prepared gDNA is clean and truly resistant.

We tested 12 more endonucleases, with different GC content in the recognition sites. None of the enzymes tested cut the viral DNA. The corresponding gel is shown in Supplemental Figure 4, and a discussion has been added in the results: "Last but not least, Halovirus HVTV-1 DNA that carries mainly $dPreQ_1$ was found to resist restriction by all enzymes tested, even those that lack guanine in the recognition site (**Figure 5E and Supplemental Figure 4**)".

3. To gain advantage, the phage genomes reported in this work "invented" modified G (7-deazaG derivatives) to protect phage genomes against restrictions. However, bacterial hosts evolve modification-dependent restriction systems to attack modified phage genomes. Examples are SRA-HNH and PvuRts1I family of modification-dependent restriction systems that can restrict 5hmC-modified phage DNA, and GmrSD family of enzymes restricting glucosylated-5hmC phage genomes. This paper highlights many hypermodified phage genomes in the phage-host biological arms race. But the next generation of arsenal- potential modification-dependent restriction systems to restrict/attack these modified DNA is missing in the discussion.
We initially chose to not speculate on the counter measures developed by the bacteria and archaea against these modifications as we did not discover any. Though, we tried to address this concern as follows in the discussion: "We can only speculate on how bacteria evolve to counteract this specific anti-restriction mechanism. As we were successful in deleting the *dpdA* gene from *Escherichia* phage CAjan using a CRISPR-Cas9 technique (see methods), we know these modifications do not provide resistance against the type II CRISPR-Cas system[4]. However, as the adaptive system of the CRISPR-Cas recognizes the nitrogen in position 7 of the guanines in the PAM[52], thus it is possible that these phages escape the degradation by the CRISPR-Cas by preventing the adaptation system to bind to its DNA. One could also imagine that other means of defense, described in recent reviews[2,3], provide an efficient protection mechanism against these phages, or that some bacteria evolved new means of defense yet to be discovered."
Other minor points:
1. The order of Figure 2B and 2C presentation is reversed. (2C presented first).
The two figures have been swapped accordingly.
2. Figure legend, page 25. Figure 7 should be Figure 6.
This has been corrected.
3. In a few places, restriction digestions carried out in 20 ul not in 20 ml.
The mistake has been corrected.
4. Phage 34. Figure 3. dPreQ0 pathway. LC-MS confirmed that it is dPreQ0.
It should be $dPreQ_0$ and has been corrected in the figure.
5. What other predicted DNA modification genes are present in the Halovirus HVTV-1 genome?
A statement has been added to page 8 in the appropriate section: "Similarly, the *Halovirus* HVTV-1 (NC_020158), presented in **Figure 3**, may have found another way to insert the modifications and should harbor either $dPreQ_1$ or dG^+ as it encodes the QueF, or QueF-like, protein."
6. Figure 4. Are the protein networks supposed to be readable by the readers? Apparently some of the nodes are too small to be eligible.
No, the individual proteins are not supposed to be readable in the SSN. The networks are just to show the clustering by color. To avoid confusion the network were done anew without the name inside of the nodes.
7. Page 22. "Washed".
Corrected
8. Supplement material.

Page 2. line 61. Species name should be in italic.
Corrected
Page 3. line 126, MgCl2, line 128, 55oC.
Corrected
Page 4. Lines 140 and 141, fix MgCl2, MgSO4, CaCl2. Line 158, fix dH2O. line 165, fix MgSO4.
Corrected
Page 5. Line 182, Fix dH2O. line 186, fix 2ul. Line 186 and 187, check oligo concentration 50mM.
Corrected
Page 9, DNA pol ge
We are not sure what the requested correction here is.

Reviewer 3

This is an interesting paper which extends recent discoveries of non-canonical bases in phage DNA, identifying three new modifications. Functionality is also demonstrated in terms of providing resistance to host restriction enzymes.

The language is quite dense and can be difficult to follow the thread of the argument. Numbered lanes are referred to in the text; e.g. lines 113 and 116), but are not numbered in Figure 2. Figures 2B and 2C seem to be reversed in the text. Similarly, bands are referred to (uncut plasmid) but not indicated in the figure so there is a bit of guesswork involved.
The lanes were numbered in Figure 2C and a white rectangle was added to guide the reader. Similarly, arrows were added on figure 2B to point at the undigested plasmids.

Paragraph (lines 142 – 147) is not clear, please re-word.
This paragraph has been rewritten as follow: "We identified a new sub-family of DpdA, renamed DpdA2, encoded by the *Vibrio* phage nt-1 by investigating genes flanking $PreQ_0$ biosynthesis genes cluster. Indeed, phage nt-1 DpdA2 (YP_008125322) is not detected when using *Enterobacteria* phage 9g DpdA as a query in PSI-BLAST. This new DpdA2 family does not possess the conserved histidine found at position 196[11]. However, some similarities with member of the TGT family were detected using HHpred, with a confidence score of 100%."

Line 152; some words missing?
This sentence has been rephrased: "Most of these viruses (163/182) were bacteriophages, while 16 of them were archaeal viruses and 3 were eukaryotic viruses".

Figure 4 is of limited value to the reader, and could be moved to Supplementary.
The information in Figure 4 is destined to show a better clustering of these proteins as they are difficult to separate by alignment. The tool used here presents an accurate way of looking at the clustering of these proteins from different origins to conclude on their functions and is important to predict G^+- or $preQ_1$-containing phages. Hence, we think it is important to keep this figure in the main text

I am not convinced about the link between modification and phage infecting pathogens - is this due to an overabundance of pathogenic bacteriophages in databases, or is it 'real'?
As explained in the text, when the whole database of sequenced phage DNA genomes was analyzed, only 9 % of them infect bacterial pathogens, compared to the 60 % found when focusing on the subset of phages encoding 7-deazaguanine modification pathways. So even though the abundance of pathogens in sequenced database is high, phages predicted to harbor 7-deazaguanines infecting bacterial pathogens are almost seven times more frequent than by chance. We realize that this reasoning might have been hard to follow in the previous text and we rephrased this passage as follows: "Interestingly, 106 of the collected phages (~ 60%) infect a host strain that is the model for a

known bacterial pathogens (**Supplemental Tables 2**), where only ~ 9% of all the dsDNA viruses from the Virus-Host database[41] infect a strain related to pathogens (data not shown), making our sample six to seven times enriched compared to a random sampling."

Lines 273-275 are speculative, not experimental results, and should be moved to the Discussion.

These lines were modified as followed: "A few bacterial hosts, such as 46 different strains of *E. coli*, *Haloarcula valismortis* and *Vibrio harveyi* 1DA3, also harbor homologs of the bacterial DpdA, that are known to modify the bacteria DNA by either $dPreQ_0$ or dADG[11]. ~~In these cases, infecting phages could be modified by the host modification machinery.~~"

This result was already discussed later in the discussion part as followed: "It is possible that in the HVTV-1 case, the host DpdA is responsible for the presence of modifications in its genome (EMA11768 in AOLQ01000002)."

REVIEWERS' COMMENTS:

Reviewer #1 (Remarks to the Author):

This paper reports on a pathway by which 7-deazaguanine bases are inserted into both phage and host DNA.

The modifications would protect the phage DNA from cleavage by host-encoded restriction endonucleases. This is very interesting work with broad implications for competition between hosts and phage.

The authors have responded appropriately to previous criticisms and suggestions.

Reviewer #2 (Remarks to the Author):

In the supplement material. Suppl. figure 6 in some places was written as Suppl. figure 64 or 46.

In Suppl. figure 1, the red arrows seem to be shifted and now point to an empty space.

The authors have addressed all the concerns that I had raised.

extra question: the specificity of the modified base insertion was not addressed in this work. It would be a topic for future study. But is there any specificity in putting in the modified base dG+, dADG, dPreQ0, or dPreQ1 in phage genomic DNA?

Nice work and congratulation!

Reviewer #3 (Remarks to the Author):

No further comments

REVIEWERS' COMMENTS:

Reviewer #1 (Remarks to the Author):

This paper reports on a pathway by which 7-deazaguanine bases are inserted into both phage and host DNA.

The modifications would protect the phage DNA from cleavage by host-encoded restriction endonucleases. This is very interesting work with broad implications for competition between hosts and phage.

The authors have responded appropriately to previous criticisms and suggestions.

Thank you for your review.

Reviewer #2 (Remarks to the Author):

In the supplement material. Suppl. figure 6 in some places was written as Suppl. figure 64 or 46.

This has been corrected.

In Suppl. figure 1, the red arrows seem to be shifted and now point to an empty space.

The red arrows point at very faint band hat are present.

The authors have addressed all the concerns that I had raised.

extra question: the specificity of the modified base insertion was not addressed in this work. It would be a topic for future study. But is there any specificity in putting in the modified base dG+, dADG, dPreQ0, or dPreQ1 in phage genomic DNA?

This is the topic of another paper we are about to submit elsewhere. Long story short, yes there is a sequence specificity for the insertion of the modifications.

Nice work and congratulation!

Thank you.

Reviewer #3 (Remarks to the Author):

No further comments

Thank you for the reviews.
